# Single-molecule fingerprinting of protein-drug interaction using a funneled biological nanopore

**DOI:** 10.1038/s41467-023-37098-4

**Published:** 2023-04-04

**Authors:** Ki-Baek Jeong, Minju Ryu, Jin-Sik Kim, Minsoo Kim, Jejoong Yoo, Minji Chung, Sohee Oh, Gyunghee Jo, Seong-Gyu Lee, Ho Min Kim, Mi-Kyung Lee, Seung-Wook Chi

**Affiliations:** 1 Disease Target Structure Research Center, Division of Biomedical Research, Korea Research Institute of Bioscience and Biotechnology (KRIBB), Daejeon, 34141 Republic of Korea; 2 Critical Diseases Diagnostics Convergence Research Center, KRIBB, Daejeon, 34141 Republic of Korea; 3 Department of Proteome Structural Biology, KRIBB School of Bioscience, University of Science and Technology, Daejeon, 34113 Republic of Korea; 4 Department of Physics, Sungkyunkwan University, Suwon, Gyeonggi 16419 Republic of Korea; 5 Center for Biomolecular and Cellular Structure, Institute for Basic Science (IBS), Daejeon, 34126 Republic of Korea; 6 Graduate School of Medical Science and Engineering, Korea Advanced Institute of Science and Technology (KAIST), Daejeon, 34141 Republic of Korea; 7 School of Pharmacy, Sungkyunkwan University, Suwon, Gyeonggi 16419 Republic of Korea

**Keywords:** Nanopores, Membrane proteins, Drug screening, Pharmacology

## Abstract

In drug discovery, efficient screening of protein-drug interactions (PDIs) is hampered by the limitations of current biophysical approaches. Here, we develop a biological nanopore sensor for single-molecule detection of proteins and PDIs using the pore-forming toxin YaxAB. Using this YaxAB nanopore, we demonstrate label-free, single-molecule detection of interactions between the anticancer Bcl-xL protein and small-molecule drugs as well as the Bak-BH3 peptide. The long funnel-shaped structure and nanofluidic characteristics of the YaxAB nanopore enable the electro-osmotic trapping of diverse folded proteins and high-resolution monitoring of PDIs. Distinctive nanopore event distributions observed in the two-dimensional (ΔI/I_o_-versus-I_N_) plot illustrate the ability of the YaxAB nanopore to discriminate individual small-molecule drugs bound to Bcl-xL from non-binders. Taken together, our results present the YaxAB nanopore as a robust platform for label-free, ultrasensitive, single-molecule detection of PDIs, opening up a possibility for low-cost, highly efficient drug discovery against diverse drug targets.

## Abstract (web summary)

Efficient screening of protein-drug interactions (PDIs) has been impeded by the limitations of current biophysical approaches. Here, the authors present a funneled YaxAB nanopore sensor which allows label-free, single-molecule detection of proteins and PDIs.

## Introduction

Current drug development suffers from high cost and low efficiency of the drug discovery process. The therapeutic efficacy of drugs is mediated by physical interaction with their cognate targets (mainly proteins). To monitor protein-drug interactions (PDIs) in vitro is essential for the drug discovery processes including drug screening, structure-activity-relationship (SAR), and mode-of-action (MOA) studies^1,2^. Although various existing biophysical approaches such as nuclear magnetic resonance (NMR), surface plasma resonance (SPR), isothermal titration calorimetry (ITC), and fluorescence have been used to directly monitor PDIs, efficient drug screening has been still impeded by severe limitations such as high-cost instrumentation, labeling or immobilization-derived inaccuracy, low-sensitivity of detection for small-molecule drugs, and limited solubility of target proteins and/or drugs^3–6^. Therefore, technological innovation is required to overcome current limitations and accelerate drug discovery in the pharmaceutical industry.

Nanopore sensing is an emerging technology for single-molecule analysis of biomolecules. Based on ionic current blockade derived from electrically drawn analytes under voltage applied across a nanoscale pore, nanopore sensors offer single-molecule resolution and ultrasensitive, label-free, real-time detection, and high-throughput analysis^7,8^. Biological nanopore sensors made with channel proteins exhibit uniform pore size, low noise, and high resolution, and are easily modifiable through protein engineering^9,10^. Although nanopore analysis of proteins is complicated by their folded structure and non-uniform charge, recent studies have attempted the application of biological nanopores to single-molecule analysis of folded proteins^11–13^. Several biological nanopores such as ClyA, PlyAB, and MspA, and solid-state nanopores including NEOtrap^14,15^ have been utilized to analyze protein conformations and protein-ligand interactions^16–18^. However, these nanopore sensors are mainly based on specific ligand-induced large conformational changes.

Although protein-protein interactions (PPIs) are promising targets, drugging them is one of the key challenges in drug discovery^19^. While more than 645,000 disease-relevant PPIs have been reported in the human interactome, only approximately 2 % of them have been targeted for drug development^20^. Thus, there is a high demand for low-cost, efficient drug screening technologies against them. The PPI between B-cell lymphoma-extra large (Bcl-xL) and Bcl-2 homology 3 (BH3) domain of Bak (Bak-BH3) is an attractive anticancer therapeutic target^21^. The Bak-BH3 is crucial for inducing cell death and its binding to Bcl-xL antagonizes the anti-apoptotic function of Bcl-xL^22,23^. Diverse BH3-mimetic compounds have been identified for anticancer drug discovery. Among them, ABT-737 is well-known as a potent small-molecule inhibitor of the Bak-BH3/Bcl-xL interaction^24^. YaxAB is a pore-forming toxin (PFT), a virulence factor produced by *Yersinia enterocolitica*, an intestinal pathogen that causes diarrhea and systemic bacteremia^25^. YaxAB was shown to be cytotoxic and involved in pathogenesis through the osmotic lysis mechanism by forming pores in mammalian cell membranes^26^.

Here, we show the single-molecule measurements of Bcl-xL and its ligand interactions using a funneled YaxAB nanopore. The current blockade and noise analyses of nanopore events reveal the ability of the YaxAB nanopore for label-free, single-molecule detection of PDIs, which could contribute to highly efficient drug discovery.

## Results

### Pore formation and nanofluidic characterization of YaxAB nanopores

**Fig. 1:**
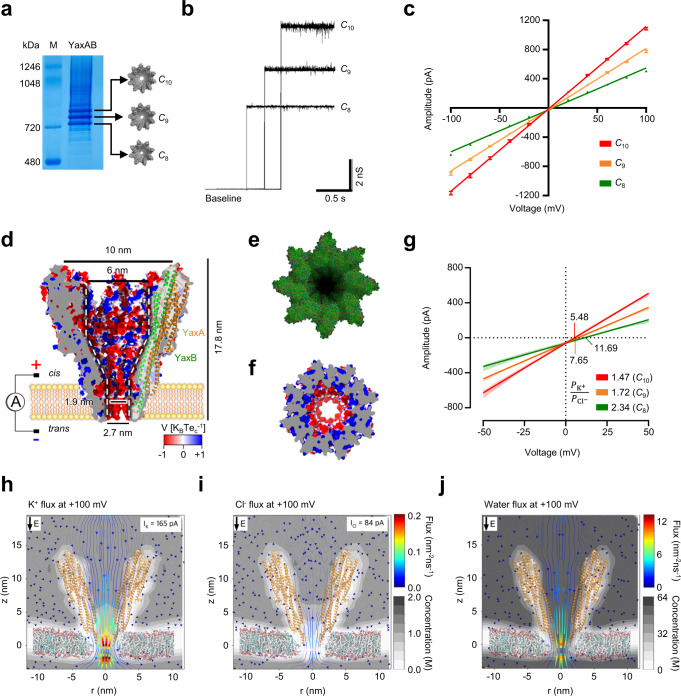
Characterization of YaxAB nanopores. Blue native gel (4–16%) electrophoresis (**a**) and single pore insertions (**b**) of YaxAB-*C*_8_, -*C*_9_, and -*C*_10_ nanopores. The representative PAGE result is presented, *n* = 3 independent replicates. Source data are provided in the Source Data file. **c** Current-voltage (I-V) plots of YaxAB-*C*_8_ (green), -*C*_9_ (orange), and -*C*_10_ (red) nanopores. Data are presented as mean ± SD, *n* = 3 independent replicates for YaxAB-*C*_8_; *n* = 6 independent replicates for YaxAB-*C*_9_; *n* = 9 independent replicates for YaxAB-*C*_10_. Source data are provided as a Source Data file. **d** Cross section of YaxAB-*C*_8_ nanopore with an overlaying illustrated representation of a YaxAB heterodimer. The electrostatic potential of the surface is computed by the adaptive Poisson-Boltzmann solver at pH 7.5 in 1 M KCl. The funnel-shaped geometry of the inner surface is shown as white dotted lines. Pore lumen, corrugated with convex 8 α-helices (**e**) and negatively charged inner surface close to the constriction (**f**). **g** Reversal potential, showing cation selectivity for all YaxAB nanopores. The reversal potential values are 11.7 ± 0.1 mV (YaxAB-*C*_8_), 7.7 ± 0.7 mV (YaxAB-*C*_9_), and 5.5 ± 0.1 mV (YaxAB-*C*_10_). All reversal potentials were measured under asymmetric salt conditions (2 M KCl on the *trans* side and 0.5 M KCl on the *cis* side) in a pH 7.5 buffer. Data are presented as mean ± SD, *n* = 3 independent replicates for each YaxAB-*C*_8_, -*C*_9_, and -*C*_10_ nanopore. Source data are provided as a Source Data file. **h–j** MD simulation of YaxAB-*C*_8_ nanopore at +100 mV voltage bias and in 1 M KCl. YaxAB nanopore exhibits a comparably strong directional K^+^ (1.03 ns^−1^, I_K_ = 165 pA) and water flow (15.71 ns^−1^) from *cis* to *trans* side against Cl^-^ flow (0.52 ns^−1^, I_Cl_ = 84 pA). Arrows represent K^+^ (**h**), Cl^-^ (**i**), and water (**j**) fluxes inside YaxAB nanopores. K^+^ (0.16 nm^−2^ · ns^−1^) and water fluxes (5.63 nm^−2^ · ns^−1^) at the constriction region were calculated at 100 mV in 1 M KCl.

Inspired by its cryo-electron microscopy (cryo-EM) structure^26^, we tested whether YaxAB toxin could be utilized as a nanopore sensor. The cryo-EM structure of YaxAB showed an α-helical pore complex assembled with the octamer to dodecamer of the YaxA-YaxB heterodimer^26^. Using native gel electrophoresis and gel extraction, we purified three types of YaxAB pore with different sizes: YaxAB-*C*_8_ (16-mer), YaxAB-*C*_9_ (18-mer), and YaxAB-*C*_10_ (20-mer) (Fig. 1a; Supplementary Fig. 1). The negative-stain EM images showed that each extracted oligomer of YaxAB-*C*_8_, YaxAB-*C*_9_, or YaxAB-*C*_10_ has 8, 9, or 10 symmetric spikes, respectively (Supplementary Fig. 2a–c). The 3D reconstruction of the YaxAB complex confirmed the *C*_8_ symmetric composition of the peripheral and interior rings of YaxA and YaxB, respectively (1:1 stoichiometry) (Supplementary Fig. 2d). Under an applied potential of 100 mV, each type of YaxAB pore showed unitary conductance: YaxAB-*C*_8_, 5.0 ± 0.1 nS; YaxAB-*C*_9_, 7.8 ± 0.2 nS; YaxAB-*C*_10_, 10.9 ± 0.2 nS (Fig. 1b and Supplementary Fig. 3). The diameters of the three pores were estimated from experimental conductance data, and were in accordance with theoretical prediction^27^ (Fig. 1b, c, Supplementary Fig. 4; Supplementary Table 1). The pore gating was intermittently observed in YaxAB-*C*_10_ and YaxAB-*C*_9_ pores, and a negligible pore gating signal was detected in YaxAB-*C*_8_ pores without any addition of analytes. Using data from the collected EM particles, we generated the structure of YaxAB-*C*_8_ with apparent *C*_8_ symmetry through three-dimensional reconstruction (Supplementary Fig. 2d). YaxAB-*C*_8_ pore exhibits an ~18 nm long, funneled internal structure with a *cis* entry of 10 nm, a *trans* entry of 2.7 nm, and a constriction with a diameter of 1.9 nm (Fig. 1d). The ‘coffee dripper’-shaped lumen of the YaxAB-*C*_8_ pore is corrugated with ‘rib’-like convex 8 α-helices (Fig. 1e–f).

To examine ion transport across the YaxAB pores, we measured ionic currents at varying applied voltages (Fig. 1c). All three pore types revealed asymmetric I-V curves. The reversal potentials (V_r_) of YaxAB pores were measured using asymmetric KCl concentration on both sides of the nanopore (*trans*/*cis*: 2 M KCl/0.5 M KCl) buffered with 10 mM Tris-HCl pH 7.5 (Fig. 1g, Supplementary Table 2). The ion selectivity of YaxAB pores was then calculated using the Goldman–Hodgkin–Katz equation (Methods, Supplementary Table 3). The strong cation selectivity (${P}_{{K}^{+}}$/${P}_{{{Cl}}^{-}}$=2.34 ± 0.03) of YaxAB-*C*_8_ nanopore (referred to as YaxAB hereafter) most likely reflects the noticeably negative charges of constriction (Fig. 1f). The high ion selectivity could induce potent electro-osmotic flow (EOF) inside the pore against the electrophoretic force (EPF)^28,29^. The molecular dynamics (MD) simulation showed local distribution profiles for ions and water inside the YaxAB nanopore, indicating an increase in K^+^ (0.16 nm^−2^ · ns^−1^) and water (5.63 nm^−2^ · ns^−1^) flux velocity closer to the constriction at the positive applied voltage (Fig. 1h-j). The K^+^ flux (0.16 nm^−2^ · ns^−1^) was stronger than Cl^-^ flux (0.04 nm^−2^ · ns^−1^) in the constriction, indicating potent EOF with *cis* to *trans* direction at the positive voltage.

### Single-molecule analysis of unlabeled proteins using YaxAB nanopores

**Fig. 2:**
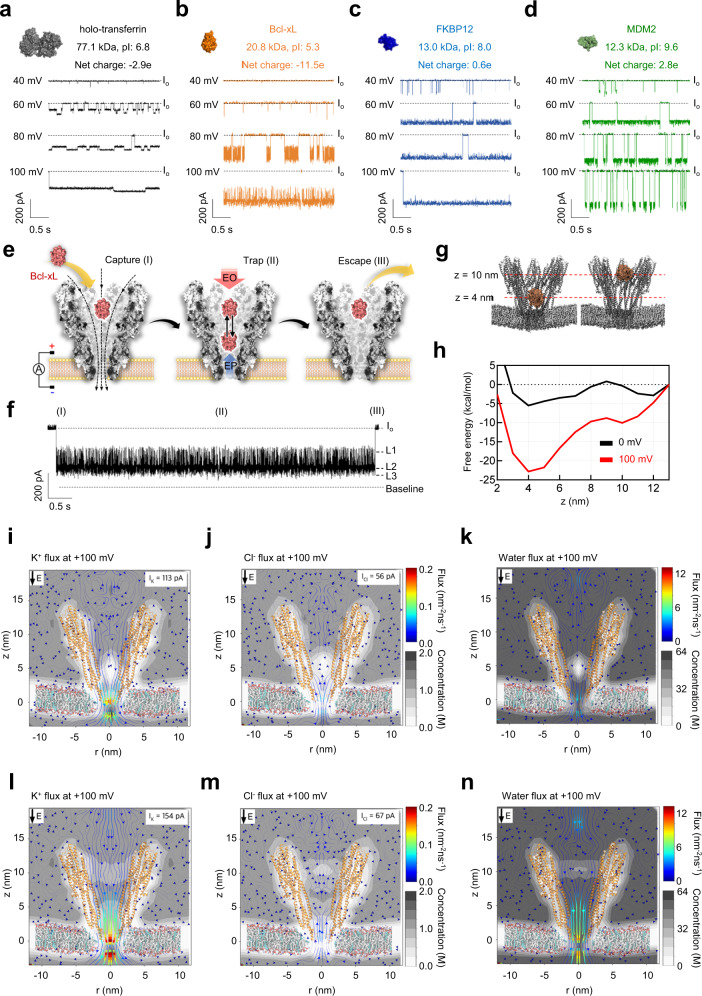
Single-molecule analysis of an unlabeled protein using YaxAB nanopores. YaxAB nanopore-based electrical recordings of proteins: holo-transferrin (holo-Tf, gray) (**a**), B-cell lymphoma-extra large (Bcl-xL, orange) (**b**), FK506 binding protein-12 (FKBP12, blue) (**c**), and Mouse double minute 2 homolog (MDM2, green) (**d**). Open pore current (I_o_) is indicated as a dotted line. Proteins were added to the *cis* side by applying a positive bias (40 to 100 mV). Current traces were filtered with a Bessel (8-pole) filter at 1 kHz. Schematic illustration of the capture-trap-escape process (**e**) and a single nanopore event of the Bcl-xL protein (**f**) using the YaxAB nanopore. **g** Simulated snapshot illustrations for Bcl-xL trapped at 4 and 10 nm positions from the membrane. “z” indicates the distance from the membrane. **h** Free energy landscapes calculated from MD simulation of free Bcl-xL within a YaxAB nanopore at the applied voltage of 100 mV. The free energy of Bcl-xL at 4 and 10 nm was −22.81 and −10.05 kcal/mol, respectively. Molecular dynamics (MD) simulation data of K^+^ (**i**), Cl^-^ (**j**), and water (**k**) fluxes through a YaxAB nanopore in the presence of Bcl-xL protein at a 4 nm position from the membrane (0.10 nm^−2^ · ns^−1^, 0.04 nm^−2^ · ns^−1^, and 2.59 nm^−2^ · ns^−1^, respectively), at 100 mV in 1 M KCl. K^+^ (0.70 ns^−1^, I_K_ = 113 pA), Cl^−^ (0.35 ns^−1^, I_Cl_ = 56 pA) and water (10.66 ns^−1^) fluxes at the constriction region were calculated at 100 mV in 1 M KCl. MD simulation data of K^+^ (**l**), Cl^-^ (**m**), and water (**n**) fluxes through YaxAB nanopore in the presence of Bcl-xL protein at a 10 nm position from the membrane (0.14 nm^−2^ · ns^−1^, 0.03 nm^−2^ · ns^−1^, and 5.42 nm^−2^ · ns^−1^, respectively), at 100 mV in 1 M KCl. K^+^ (0.96 ns^−1^, I_K_ = 154 pA), Cl^−^ (0.42 ns^−1^, I_Cl_ = 67 pA) and water fluxes (15.71 ns^−1^) at the constriction region were calculated at 100 mV in 1 M KCl.

To assess the sensing capability of the YaxAB nanopore, we attempted to capture four proteins of different charges and sizes: holo-transferrin (9.2 × 6.4 × 4.9 nm), Bcl-xL (4.6 × 4.6 × 3.5 nm), FKBP12 (4.5 × 3.5 × 3.0 nm), and MDM2 (3.0 × 3.7 × 3.8 nm). To test whether the proteins are still folded in the nanopore condition, we further performed circular dichroism (CD) experiments of the proteins in the nanopore measurement buffer including 1 M KCl. As shown in Supplementary Fig. 5, no structural change of the proteins was observed in the nanopore buffer condition. All the proteins were trapped inside the nanopore by EOF when introduced to the *cis* side under positive voltages (Fig. 2a–d). Using EOF, the YaxAB nanopore was capable of capturing diverse folded proteins with a broad range of mass (12~77 kDa), irrespective of their net charges. The dwell time and current blockade of the trapped Bcl-xL were significantly increased with the increment of voltage (Supplementary Figs. 6 and 7) because the direction of the electric field was the same as that of EOF-trapping by YaxAB nanopores (from *cis* entry to *trans* side). Despite their small volumes, FKBP12 and MDM2 caused larger current blockages than the other proteins. Due to their neutral or positive charges at pH 7.5, they could undergo weaker repulsive EPF (from *trans* to *cis* entry) than the other proteins, which allows their movements to a deeper site with higher ion density within the YaxAB nanopore.

To investigate the effect of net charge on the capture rate and trapping event frequency of a protein by the YaxAB nanopore, we measured the capture rate (1/τ_on_) and the reciprocal of dwell time (1/τ_off_) of wild-type (net charge: −11.5e) and mutant Bcl-xL (Bcl-xL_R100E,R103E) with additional negative charges (net charge: −14.8e) (Supplementary Fig. 8). The capture rate (1/τ_on_) of Bcl-xL_R100E,R103E as a function of voltage bias was almost 3-fold lower than that of wild-type Bcl-xL. Additionally, the 1/τ_off_ value of Bcl-xL_R100E,R103E was higher than that of wild-type Bcl-xL, while they showed a voltage-dependent reduction in 1/τ_off_. This substantial reduction in both capture rate and dwell time (τ_off_) could be attributed to stronger repulsive EPF (from *trans* to *cis* entry) imposed on Bcl-xL_R100E,R103E with more negative charges than wild-type Bcl-xL, which is consistent with the previously reported result^30^. These results indicated that the net charge of Bcl-xL protein could influence the balance between EOF and EPF within the YaxAB nanopore, thereby changing its capture rate and trapping event frequency.

From the nanopore measurements, we found that an unlabeled Bcl-xL molecule trapped inside the YaxAB nanopore generated characteristic nanopore events with frequent current spikes of multi-level amplitudes (Fig. 2b, e, f). Based on these current patterns, we propose a molecular model of three stages for the single nanopore event of Bcl-xL (Fig. 2e). At stage I, a single Bcl-xL molecule is captured inside the pore by the *cis-*to-*trans* EOF. At stage II, the trapped Bcl-xL molecule generates multiple current blockade levels (L1-L3) with short spikes. The multi-level current blockades may be attributed to oscillating translational motions of a Bcl-xL molecule inside the pore, which is governed by a balance between the capture-inducing EOF and -opposing EPF^31^. At stage III, the blockade current is recovered to an open pore current (I_o_) level when Bcl-xL escapes from the pore. Similarly, characteristic nanopore events with multiple current levels were observed in FKBP12, MDM2, and holo-transferrin trapped by the YaxAB nanopores (Fig. 2a, c, d).

From the MD simulation, the calculated free energy landscape of the trapped Bcl-xL molecule showed double minima of free energy at the distances (z) of 4 and 10 nm from the membrane (Fig. 2g, h). This suggests that, under the energy minima, a Bcl-xL molecule undergoes oscillating movements among multiple residence sites (R1-R3) where the transient protein residence induces varying current blockade levels (L1-L3) (Fig. 2f). The lowest free energy value of Bcl-xL, observed at z = 4 nm suggests that the protein is located preferentially at this position once trapped within the YaxAB nanopore. The MD simulation predicted that the K^+^ ion and water fluxes in the constriction would be considerably reduced upon Bcl-xL trapping at z = 4 nm (Fig. 2i–k) compared to those of the empty YaxAB nanopore. Accordingly, due to the weakened EOF, Bcl-xL would move toward the *cis* entry (z = 10 nm). The MD simulation data showed that Bcl-xL trapped at z = 10 nm experiences higher fluxes of K^+^ and water than at z = 4 nm (Fig. 2l–n), suggesting that Bcl-xL migrates back to the *trans* side (z = 4 nm) due to the recovered EOF. To understand the origin of the observed multiple current levels and to quantify the dynamic exchange between the two energy minima, we performed 1D Brownian dynamics simulations without z-axis restriction at 30 mV voltage bias (Supplementary Fig. 9). The thermal fluctuation allowed Bcl-xL to move between z = 4 nm and z = 10 nm sites. Similar to the experimental results, the Brownian dynamics simulation generated a current trace as well as a positional trace (Supplementary Fig. 9). These are consistent with the observed free energy landscape (Fig. 2h) as previously reported^32^.

To understand the effect of net charge on Bcl-xL movement within a YaxAB nanopore, we performed nanopore experiments with two Bcl-xL variants with different net charges (Bcl-xL_E31K,E36K: −7.4e; Bcl-xL_R100E,R103E: −14.8e) (Supplementary Fig. 10). Bcl-xL_R100E,R103E showed a substantial increase in event frequency of L1 with the smallest current blockade (Supplementary Fig. 10b). Due to its additional negative charges, Bcl-xL_R100E,R103E could undergo stronger repulsive EPF (from *trans* to *cis* entry) than wild-type Bcl-xL, which resulted in dominant residence at the shallower site. In contrast, Bcl-xL_E31K,E36K exhibited increased event frequency of L3 with the largest current blockade (Supplementary Fig. 10b). Bcl-xL_E31K,E36K could be subject to weaker repulsive EPF than wild-type Bcl-xL, inducing its movement to the deeper site. In addition, we performed voltage-dependent nanopore experiments with them (under 60, 80, and 100 mV). With higher voltages engaged, wild-type Bcl-xL and the variants showed noticeable decreases of I_res_ in all the current levels (L1-L3) (Supplementary Fig. 10c), indicating that they moved to deeper sites within the nanopore. This net charge- or voltage-dependent movement of protein within the YaxAB nanopore is consistent with the previously reported results^13,32^. Taken together, these results suggest that EPF and EOF control the movement of a protein between two energy minima in the YaxAB nanopore and the movement leads to different current levels.

### Current blockade-based analysis of protein-ligand interactions using YaxAB nanopores

**Fig. 3:**
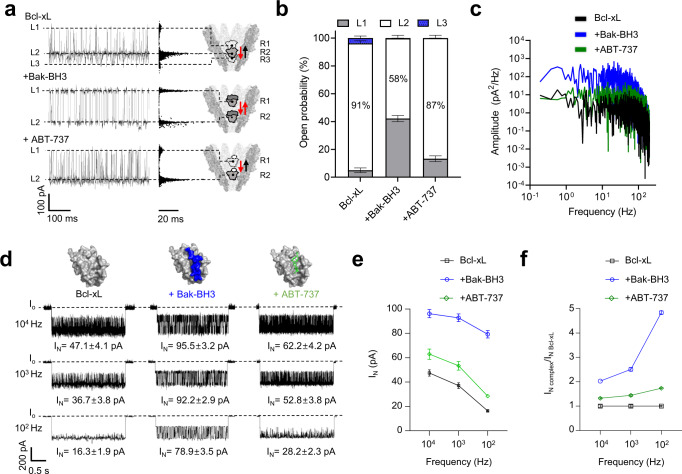
Current blockade and noise analyses of protein-ligand interactions using YaxAB nanopores. **a** Multi-level current blockades, scatter plots (I_res_ vs. duration), and models for residence sites of free Bcl-xL, Bcl-xL/Bak-BH3 (1:2), and Bcl-xL/ABT-737 (1:2) complexes. Based on the free energy calculation of Bcl-xL and experimental data of multi-level current blockades, residence sites of analytes were modeled within the YaxAB nanopore. **b** Stacked columns for the open probability of L1-L3 levels measured from current blockades of free Bcl-xL, Bcl-xL/Bak-BH3, and Bcl-xL/ABT-737 complexes. Data are presented as mean ± SD, *n* = 3 independent replicates. L1 (4.98 ± 1.65%), L2 (91.25 ± 1.89%), and L3 (3.77 ± 1.47%) for free Bcl-xL; L1 (42.13 ± 2.16%), L2 (57.71 ± 2.15%), and L3 (0.16 ± 0.03%) for Bcl-xL/Bak-BH3 complex; L1 (13.29 ± 2.07%), L2 (86.66 ± 2.07%), and L3 (0.05 ± 0.03%) for Bcl-xL/ABT-737 complex. Source data are provided as a Source Data file. **c** Power spectral density analysis of free Bcl-xL (black), Bcl-xL/Bak-BH3 (blue), and Bcl-xL/ABT-737 (green) complexes. **d** Current noise (I_N_)-analysis of free Bcl-xL, Bcl-xL/Bak-BH3 (1:2), and Bcl-xL/ABT-737 (1:2) complexes with varying filter cut-off frequency (10^2^, 10^3^, and 10^4 ^Hz). I_N_ values (**e**) and I_N_ ratios of complexed vs. free Bcl-xL (**f**) curves as a function of filter frequency for free Bcl-xL, Bcl-xL/Bak-BH3, and Bcl-xL/ABT-737 complexes. Data are presented as mean ± SD, *n* = 3 independent replicates. Source data are provided as a Source Data file.

Next, we analyzed the interactions of Bcl-xL with the Bak-BH3 peptide and a small-molecule drug, ABT-737, using YaxAB nanopores. As shown in Fig. 2f and 3a, trapping a free Bcl-xL molecule inside the YaxAB nanopore generated three current levels (L1-L3). With an increase in applied voltage, residual current (I_res_) values of all the current levels and duration of the L1 event decreased, and durations of L2 and L3 events increased, implying that free Bcl-xL moves closer to the constriction under higher voltage (Supplementary Fig. 7). On the other hand, the Bcl-xL/Bak-BH3 complex predominantly induced two current levels (L1 and L2) with similar durations and a negligible L3 level, indicating occupancy of the R1 and R2 sites with similar probability (Fig. 3a, b). YaxAB nanopore sensing also showed the voltage-dependent reduction in I_res_ for the Bcl-xL/Bak-BH3 peptide complex (Supplementary Fig. 11). Upon complexation of Bcl-xL with ABT-737, two current levels (L1 and L2) were detected, with a slight increase of L1 event frequency compared to that observed for free Bcl-xL (Fig. 3a, b). These results showed that YaxAB nanopores can sensitively detect the interactions of Bcl-xL with the Bak-BH3 peptide and ABT-737.

Our finding of clear differences in current distribution among the protein complexes is applicable to monitoring of PPI inhibition by small-molecule drugs using YaxAB nanopores. When we titrated the 1:2 Bcl-xL/Bak-BH3 complex with ABT-737, the probability of the L1 level decreased, while that of the L2 level significantly increased compared to that of the Bcl-xL/Bak-BH3 complex (Supplementary Fig. 12). Upon titration to a molar ratio of 1:2:5 (Bcl-xL:Bak-BH3:ABT-737), the probability of the L2 level was same as that of the 1:2 Bcl-xL/ABT-737 complex, indicating that ABT-737 fully displaced the Bak-BH3 peptide bound to Bcl-xL. Therefore, current blockade-based analysis using YaxAB nanopores can be effectively used to probe PPIs and their small-molecule drug inhibition at the single-molecule level.

### Current noise (I_N_)-based analysis of protein-ligand interactions using YaxAB nanopores

In addition to current blockade analysis, current noise (I_N_)-based analysis was employed to discriminate Bcl-xL/ligand complexes from free Bcl-xL using YaxAB nanopores (Fig. 3c–f). The I_N_ values were computed by analyzing power spectral density (PSD) data derived from nanopore events using Clampfit 11.2 software (Methods). The I_N_ values of free Bcl-xL and the Bcl-xL/ligand complexes were dependent on filtering condition variances (100 to 10,000 Hz) (Fig. 3d). By filtering at a particular low-frequency (100 Hz), I_N_ values provoked substantial differences among three protein analytes: free Bcl-xL, I_N_ = 16.3 ± 1.9 pA; Bcl-xL/Bak-BH3 complex, I_N_ = 78.9 ± 3.5 pA; Bcl-xL/ABT-737 complex, I_N_ = 28.2 ± 2.3 pA (Fig. 3c–f). The varying noise levels of the analytes may be attributed to the detectable difference among them in orientation and strength of dipole moment (Supplementary Fig. 13 and Supplementary Table 4).

Furthermore, we performed I_N_-based, dose-dependent quantitative analysis on the interactions of Bcl-xL with the Bak-BH3 peptide and small-molecule drugs, ABT-737 and A-1331852, yielding binding affinities in the order of A-1331852 > ABT-737 > Bak-BH3 peptide (*K*_D_ = 66 ± 10, 38 ± 8 nM, and 19 ± 4 nM, respectively) (Supplementary Figs. 14–17). In the bulk experiments, the *K*_D_ values of Bcl-xL interactions with Bak-BH3 peptide, ABT-737, and A-1331852 were determined to be 340 ± 30 nM from NMR^23^, 106 ± 17 and 13 ± 2 nM from SPR (Supplementary Fig. 18), respectively. This difference in the *K*_D_ values may arise from the intrinsic distinction between single-molecule nanopore sensing and ensemble experiments. Kinetic rate constants can be most directly measured using the single-molecule nanopore method, and its superior sensitivity enables the measurement of protein-ligand binding at even low concentrations that could be hardly detected using SPR. As shown in a previous study, the kinetics of ligand binding may be affected by the applied potential and the pore confinement imposed on trapped proteins^16^. In the case of SPR, immobilization of proteins to the sensor chip may sterically hinder analyte binding or lead to a change in local analyte concentration at the sensor surface^33^. Overall, detailed I_N_ analysis using YaxAB nanopores allowed clear discrimination of protein-ligand complexes from free proteins, most remarkably a small-molecule drug complex without any significant conformational change of protein.

### Single-molecule fingerprinting of PDIs using YaxAB nanopores

**Fig. 4:**
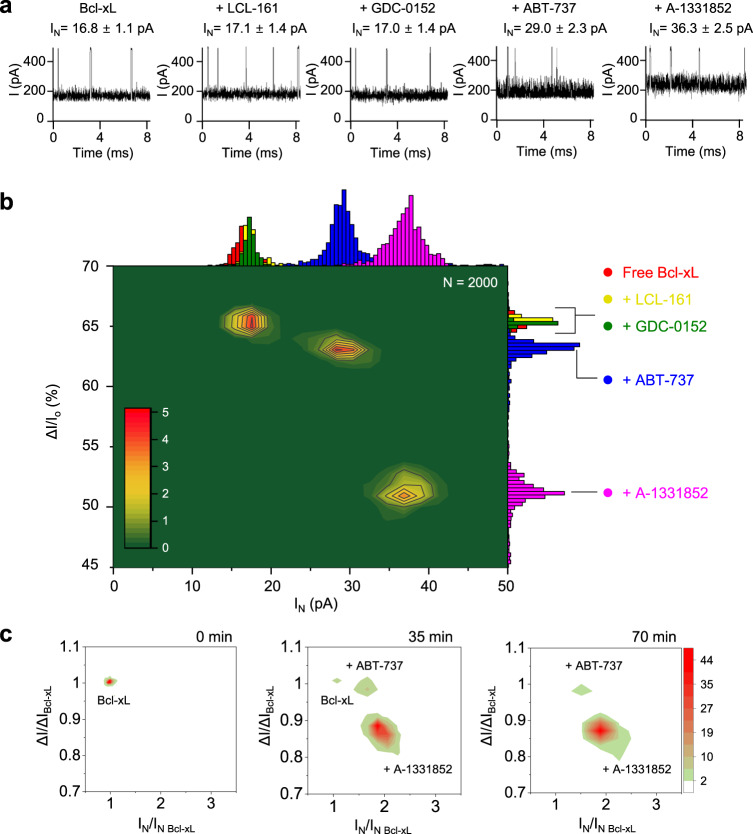
Single-molecule detection of interactions between Bcl-xL and small-molecule drugs using YaxAB nanopores. **a** Representative ionic current traces for Bcl-xL in the absence or presence of non-binders (LCL-161 and GDC-0152) and strong binders (ABT-737 and A-1331852). Each small-molecule was premixed with Bcl-xL at a 1:10 molar ratio and then added to the *cis* chamber. Nanopore events were recorded in 1 M KCl at pH 7.5 with +100 mV applied voltage on the *cis* side. Values of current blockade (ΔI/I_o_) and current noise (I_N_) were obtained after additional filtration using a 100 Hz Bessel (8-pole) filter. **b** 2D density contour plot of ΔI/I_o_-versus-I_N_ was generated by combining the data from individual YaxAB nanopore measurements for Bcl-xL in the absence or presence of each small-molecule drug (LCL-161, GDC-0152, ABT-737, or A-1331852). Color-coded marginal histograms represent free Bcl-xL, red; LCL-161, yellow; GDC-0152, green; ABT-737, blue; A-1331852, pink. **c** 2D density contour plots for real-time monitoring using the current recording of YaxAB nanopores after simultaneous treatment of Bcl-xL with a mixture of compounds (LCL-161, GDC-0152, ABT-737, and A-1331852). The measured ΔI and I_N_ values are normalized by ΔI_Bcl-xL_ and I_N Bcl-xL_ of free Bcl-xL, respectively.

Based on the observed normalized current blockade (ΔI/I_o_) and I_N_ differences among the analytes, we further tested whether YaxAB nanopores can distinguish small-molecule Bcl-xL binders from non-binders. Prior to PDI analysis, we measured the open conductance of the YaxAB nanopore with a mixture of non-specific small-molecule ligands (Supplementary Fig. 19a). The current traces showed no difference in the absence or presence of the small-molecule ligand mixture, indicating that YaxAB nanopore does not interact with the non-specific ligands tested. After trapping of Bcl-xL within the YaxAB nanopore, the addition of small-molecule non-binders (LCL-161, GDC-0152, Birinapant, and Phentolamine) induced no detectable difference in the current traces (Supplementary Fig. 19b–e), indicating that there was no interference with the target protein signal. For example, LCL-161 and GDC-0152 showed essentially the same ΔI/I_o_ and I_N_ values (ΔI/I_o_ = 65.3 ± 0.6 and 65.0 ± 0.9%, I_N_ = 17.1 ± 1.4 and 17.0 ± 1.4 pA, respectively) as that of free Bcl-xL (Fig. 4a). In contrast, the addition of strong Bcl-xL binders (ABT-737 or A-1331852)^34^ provoked more than 1.7-fold higher current noise levels (I_N_ = 29.0 ± 2.3 and 36.3 ± 2.5 pA, respectively) than those of free Bcl-xL (ΔI/I_o_ = 65.2 ± 0.5% and I_N_ = 16.8 ± 1.1 pA) along with changes in ΔI/I_o_ (ΔI/I_o_ = 63.0 ± 0.8 and 51.1 ± 2.0%, respectively). Combining the data from individual measurements, a two-dimensional (2D) ΔI/I_o_-versus-I_N_ density plot showed that the event distributions of non-binder/Bcl-xL mixtures are largely overlapped with those of free Bcl-xL (Fig. 4b). In contrast, strong binder/Bcl-xL complexes were clearly distinguished from free Bcl-xL as two separate peaks, or “drug fingerprints”. Indeed, YaxAB nanopores could sensitively distinguish different small-molecule drugs bound to the same protein, which provided a proof-of-concept for single-molecule fingerprinting of PDIs using a biological nanopore sensor.

To examine whether this nanopore sensing could be applied to drug screening with a mixture of multiple compounds, we monitored PDIs in real-time using a current recording of YaxAB nanopores. After simultaneous treatment of Bcl-xL with a mixture of compounds, including non-binders and the strong-binders, the event populations of free Bcl-xL considerably decayed to 2.5% in 35 min and 0.7% in 70 min (Fig. 4c). In contrast, the event populations of Bcl-xL/strong-binder complexes appeared gradually, eventually leading to the existence of two distinctive event populations of A-1331852 and ABT-737 complexes after 70 min (Fig. 4c). The relative percentage of Bcl-xL/ABT-737 events increased to 10.7% in 35 min, then decreased to 6.3% in 70 min, whereas that of Bcl-xL/A-1331852 events continually increased to 86.8% and 93.1% at these respective time point. Taken together, these data demonstrated that YaxAB nanopores can be used for a single-molecule-level drug screening for Bcl-xL with high selectivity.

**Fig. 5:**
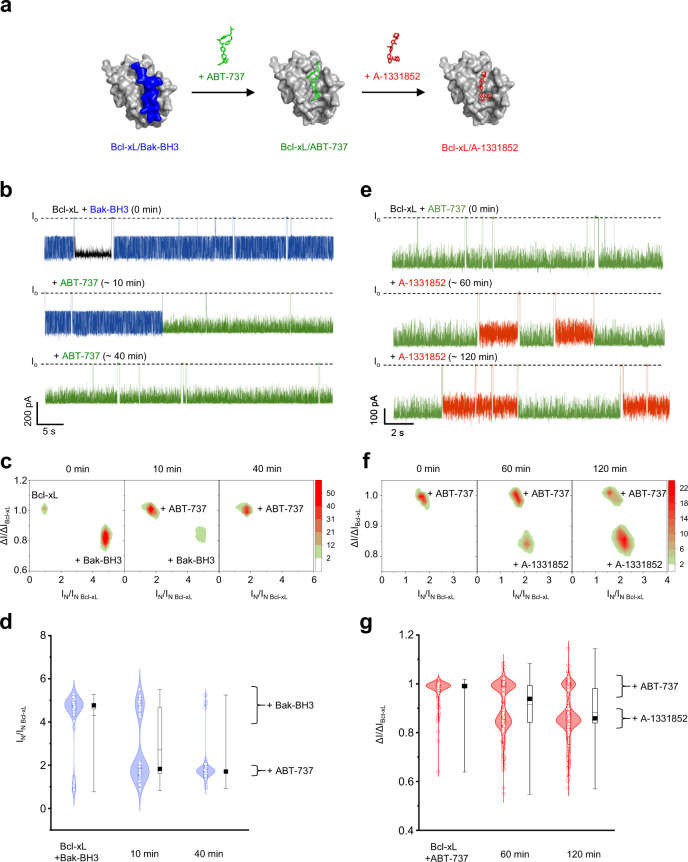
YaxAB nanopore measurements of drug competition for binding to Bcl-xL. **a** Schematic illustration of small-molecule drug competition. **b** Representative ionic current traces for the Bcl-xL/Bak-BH3 complex obtained from time-course recording upon the titration with ABT-737 at a molar ratio of 1:2:2 (Bcl-xL:Bak-BH3:ABT-737). Blue and green current traces indicate nanopore events of Bcl-xL/Bak-BH3 and Bcl-xL/ABT-737 complexes, respectively. **c** 2D density contour plots for real-time monitoring of competition between the Bak-BH3 peptide and ABT-737 for Bcl-xL binding. **d** Time-course monitoring of competition between Bak-BH3 peptide and ABT-737 for Bcl-xL binding. Data are presented as violin plots with jitter points in a combination of kernel smoothed density plots, where the box plots include the first and third quartiles 25–75%, the median (black square), dashed lines represent mean (center line), maxima and minima (whiskers). The number of events is *N* = 466 at 0 min; *N* = 180 at 10 min after the addition of ABT-737; *N* = 421 at 40 min after the addition of ABT-737. **e** Representative ionic current traces for the Bcl-xL/ABT-737 complex obtained from time-course recording upon the titration with A-1331852 at the molar ratio of 1:2:2 (Bcl-xL:ABT-737:A-1331852). Green and red current traces indicate nanopore events of Bcl-xL/ABT-737 and Bcl-xL/A-1331852 complexes, respectively. **f** 2D density contour plots for real-time monitoring of competition between ABT-737 and A-1331852 for Bcl-xL binding. **g** Time-course monitoring of the competition between ABT-737 and A-1331852 for Bcl-xL binding. Data are presented as violin plots with jitter points in a combination of kernel smoothed density plots, where the box plots include the first and third quartiles 25–75%, the median (black square), dashed lines represent mean (center line), maxima and minima (whiskers). The number of events is *N* = 319 at 0 min; *N* = 1330, 60 min after the addition of A-1331852; *N* = 2141 at 120 min after the addition of A-1331852.

Further, we conducted drug competition experiments, in which pre-formed Bcl-xL/Bak-BH3 and Bcl-xL/ABT-737 complexes were treated with ABT-737 and A-1331852, respectively (Fig. 5a). Upon titration with ABT-737, event populations of the Bcl-xL/Bak-BH3 complex were replaced with those of the Bcl-xL/ABT-737 complex (Fig. 5b). In the 2D ΔI/I_o_-versus-I_N_ plots, event populations of the Bcl-xL/Bak-BH3 complex gradually shifted the position over time to those of the newly formed Bcl-xL/ABT-737 complex (Fig. 5c, d). Forty minutes after the addition of ABT-737, only Bcl-xL/ABT-737 event populations remained, implying that ABT-737 fully displaced the Bak-BH3 peptide bound to Bcl-xL. Upon addition of A-1331852, event populations of the Bcl-xL/ABT-737 complex were largely substituted with those of the Bcl-xL/A-1331852 complex, indicating that the small-molecule drugs compete with each other for binding to the same site on Bcl-xL (Fig. 5e–g). Collectively, these data suggested the potential of “SAR-by-nanopore” analysis for screening the drug-binding activity and mapping the binding site at the single-molecule level.

To test whether YaxAB nanopore analysis can be also applicable to other targets, we further performed nanopore experiments with FKBP12 and holo-transferrin. We found that the interaction between FKBP12 and its small-molecule drug, FK506^35^ induced noticeable changes in current blockade levels (Supplementary Fig. 20). Although free FKBP12 showed three distinct current blockade levels (L1-L3), FKBP12/FK506 complex revealed two major current blockade levels (L2 and L3) with a significant decrease in the event frequency of L1. This difference between free FKBP12 and FKBP12/FK506 complex indicated that YaxAB nanopores can be used to detect the PDIs of FKBP12. Furthermore, we monitored the interaction between holo-transferrin (molecular weight: 77.1 kDa) and its small-molecule ligand, oxaliplatin (molecular weight: 397.3 Da)^36^ using YaxAB nanopores. From the current blockade-based analysis, free holo-transferrin showed the current pattern with two major current blockade levels of L1 and L2 with similar event frequencies. In contrast, the holo-transferrin/oxaliplatin complex provoked a distinctive increase in the intensity of L2 current blockade (Supplementary Fig. 21). This marked change in current blockade level induced by drug binding indicated that YaxAB nanopores can obviously detect PDIs even at a high molecular weight ratio of protein to drug (~194).

Generally, high ligand concentration is required for efficient drug screening. To test PDI detection at high ligand concentrations, we performed nanopore experiments with low-affinity Bcl-xL binders, Quercetin (*K*_D_ = 1.1 μM)^37^, and Bax-BH3 peptide (*K*_D_ = 13 μM)^23^. In the presence of a small-molecule inhibitor, Quercetin, and Bax-BH3 peptide at high concentrations, we detected current patterns (P2 state, Supplementary Figs. 22-23) distinct from those of free Bcl-xL (P1 state), indicating their specific binding to Bcl-xL. These results suggest that the YaxAB nanopore sensing method has potential application in the detection of protein-ligand interactions with affinity in the low micromolar range. To further test whether DMSO can be used for YaxAB nanopore analysis, we performed nanopore measurements in the presence of DMSO. We found that Bcl-xL can be detected by YaxAB nanopores within a range of 0-5% DMSO (Supplementary Fig. 24). Furthermore, YaxAB nanopores permitted the PDI analysis of Bcl-xL and its two small-molecule drugs (ABT-737 and A-1331852) even in the presence of 5% DMSO (Supplementary Fig. 24b, d).

## Discussion

In this study, YaxAB nanopore sensors exhibit the ability to analyze unlabeled folded proteins and PDIs with high resolution and single-molecule sensitivity. The sensing capabilities of the YaxAB nanopore may arise from its structure and nanofluidic characteristics. (i) The YaxAB nanopore forms a substantially long (~18 nm) funnel-shaped structure, the lumen of which is corrugated with convex 8 α-helices. Owing to this long-funneled geometry of the YaxAB nanopore, ion fluxes are higher closer to the pore constriction, as supported by MD simulation, suggesting that the protein blockade signal is more sensitively influenced by the position of the protein within the pore. Compared with cylindrical nanopores, this could result in an increase in the current blockade difference between protein residence sites in the YaxAB nanopore, which can be seen from the current blockade (ΔI/I_o_) difference of ~38% between residence sites (Fig. 3a). (ii) Moreover, even closing off the pore at high voltage led to a characteristic residual current without clogging (L2 I_res_ = ~35% at 100 mV; Supplementary Fig. 7), which might be attributed to ionic current flow through concave gaps in the corrugated YaxAB pore lumen. Taken altogether, these findings provide insights into how YaxAB nanopore sensors can monitor PDIs with high resolution. (iii) Owing to highly negative charges clustered near the constriction, YaxAB nanopores exhibit strong ion selectivity. The resultant potent EOF inside the pore allows the YaxAB nanopore to trap various folded proteins irrespective of their net charge. (iv) Notably, smaller protein molecules such as MDM2 and FKBP12 were shown to induce larger current blockages in the YaxAB nanopore. Weaker repulsive EPF (from *trans* to *cis* entry) imposed on the positive-charged proteins could result in their higher accessibility to the narrow pore constriction. This illustrates a specific sensing feature of a funnel-shaped biological nanopore, where current blockage depends on not only the molecular size but also the charge of a molecule.

Previously, it was shown that small-molecule ligand bindings of trapped proteins could be detected using cylindrical ClyA nanopores^17,18,38^. In a ClyA nanopore, the ligand-induced conformational change of protein results in movement in its residence site within the pore, thereby inducing differential residual currents^16^. However, there are still limitations in that ClyA nanopores have been used to indirectly probe ligand binding through an accompanied conformational change of proteins and to trap only small-sized proteins (<42 kDa)^16^. In contrast, based on the oscillating movement of a protein within its long funneled geometry, a YaxAB nanopore has a much higher sensing resolution than those of cylindrical pores; Although a ClyA nanopore could not distinguish glucose from galactose bound to GBP^18,39^, a YaxAB nanopore can sensitively discriminate small-molecule drugs (ABT-737 and A-1331852) bound to Bcl-xL independently of any significant conformational change. Moreover, the funneled YaxAB nanopore with a wider entry has the potential to analyze a larger size of proteins (~77 kDa, holo-transferrin in this study) than those trapped by a ClyA nanopore.

The different drug bindings make a significant distinction in dipole characteristics (orientation and strength of dipole moment) among the complexes (Supplementary Fig. 13 and Supplementary Table 4). This may lead to a differential effect on the oscillating movement of the complexes between different sites within the YaxAB nanopore. The resultant changes in the dynamic motion of the complexes could be sensitively detected by current or noise as previously described^16,40,41^. In this study, we showed that I_N_ as well as current blockades can be employed for analyzing protein-ligand interactions using YaxAB nanopores. The subevent information of proteins obtained from noise-based nanopore analysis has been used to analyze ligand binding, conformational change, and post-translational modification^16,42–44^. The I_N_ of the nanopore event reflects the intrinsic protein dynamics including tumbling motion inside the pore^16,45^, and may be related to overall shape, charge, volume, dipole moment, and rotational diffusion coefficient of proteins^45,46^. In particular, the flicker (1/*f*) noise, a dominant source of I_N_ at low frequency (<100 Hz), is associated with slow fluctuations in the number and mobility of the charge carriers including the change in dipole moment^47^. Thus, such a big difference among the protein analytes was observed in the low-frequency noise (I_N_) rather than total noise. Taken together, even in the absence of any significant conformational change, the binding of small-molecule drugs to proteins could induce a variation of dipole moments, thereby resulting in noticeable I_N_ differences among protein-drug complexes. Through current blockade and noise analyses using YaxAB nanopores, distinctive event distributions on the 2D ΔI/I_o_-versus-I_N_ density plot allow for “drug fingerprinting-by-nanopore”, which enables us to sensitively discriminate different small-molecule drugs bound to the same target protein.

In the present study, we demonstrated single-molecule-based PDI analysis using YaxAB nanopore sensors. This biophysical measurement observed at a single-molecule level could not be achieved with current PDI detection methods. Thus, YaxAB nanopore sensors offer distinguished advantages over ensemble-averaging-based conventional approaches: (i) single-molecule-based ultrahigh sensitivity of YaxAB sensors enables PDI detection with ultralow amounts of sample (~picomole-level), which permits the analysis with poorly soluble small-molecule compounds and proteins (*vs*. the large amount (~mg) of highly soluble samples are required for NMR^6^ and ITC^4^); (ii) label-free and low-cost implementation (*vs*. time-consuming and high-cost labeling or immobilization is required for fluorescence-based spectroscopy^5^, NMR, or SPR^3^); and (iii) real-time quantitative analysis for probing transient or weak-affinity PDIs, which could be hardly detected by ensemble experiments due to averaging-out of minor populations.

In combination with Bcl-xL data, our results on FKBP12 and holo-transferrin showed that the YaxAB nanopore can be used to analyze PDIs for various drug targets rather than specific targets. Additionally, the trapping ability of YaxAB nanopores was demonstrated using proteins with a wide range of molecular weight (12~77 kDa) and net charge (−11.5e~2.8e) (Fig. 2a-d). These results indicated the potential of the YaxAB nanopores in general PDI assay for well-verified drug targets as well as undruggable PPI targets. To estimate the upper limit of molecular weight of a protein, we further performed the YaxAB nanopore measurements with plasminogen (90 kDa; 5.2 × 7.3 × 10.6 nm) and PI3 kinase (126 kDa; 7.1 × 7.8 × 10.0 nm), but could not detect stable trapping of them by the YaxAB nanopores. Our observation of trapping of holo-transferrin (77 kDa; 4.9 × 6.4 × 9.2 nm) by the YaxAB-*C*_8_ nanopores suggested the upper limit of molecular weight of a protein to be approximately 77 kDa, which is consistent with the internal diameter (~6 nm) of the pore lumen at *cis* entry.

We found that the YaxAB nanopore system is compatible with the use of 0–5% DMSO, which is often used for poorly soluble small-molecule compounds. Although the current YaxAB nanopore system does not appear to be sufficiently stable in the presence of detergents (less stable at >0.001% Tween20), further implementation of stable polymer membranes will enable nanopore sensing with detergents at high concentrations required to avoid aggregation or non-specific binding of compounds. Further, high-throughput sensing of nanopore sensors would accelerate their application to drug screening. Although there were recent advances such as nanopore sequencing with > 512 channels (by Oxford Nanopore Technologies)^48,49^ and high-throughput automated patch clamp^50^, nanopore-based drug screening awaits further technical development of automated high-throughput sensing.

We present a YaxAB nanopore sensor for analyzing unlabeled folded proteins and protein-ligand interactions. Our results revealed that YaxAB nanopores can be a valuable platform for label-free, ultrasensitive, and single-molecule-level detection of PDIs, indicating great potential for drug discovery applications including drug screening, SAR, and MOA analyses. Compared with conventional ensemble-averaging-based techniques that require labeling, immobilization, expensive instruments, and time-consuming or labor-intensive procedures, YaxAB nanopore sensors could greatly reduce cost and increase the speed of drug discovery, especially when coupled with high-throughput analysis. Particularly, the “drug fingerprinting-by-nanopore” with single-molecule sensitivity will be used for drug screening with an ultralow amount of samples, which is greatly beneficial for undruggable or insoluble targets. Furthermore, the YaxAB nanopores could be utilized to probe drug-induced subtle allosteric conformational or dynamics changes in single-molecule proteins with ultrahigh-resolution, which could advance their beneficial use in drug discovery applications including HTS, fragment-based drug design, and SAR analysis for lead optimization. Finally, our findings may open up a route for low-cost, highly efficient drug discovery against diverse drug targets, including undruggable PPI targets.

## Methods

### Reagents

Chemicals in the nanopore measurement buffer were purchased from Sigma-Aldrich (St. Louis, USA). Lipids and detergents were purchased from Avanti Polar Lipids (Alabaster, AL, USA) and Anatrace (Maumee, OH, USA), respectively. Bak-BH3 peptides (NH_2_- GQVGRQLAIIGDDINR-COOH) were purchased from Peptron Inc. (Daejeon, Republic of Korea). Small-molecule drugs, ABT-737 (813.43 g/mol, Cayman Chemical, Ann Arbor, USA), LCL-161 (500.63 g/mol, Selleckchem, Houston, USA), GDC-0152 (498.64 g/mol, Selleckchem), A-1331852 (658.81 g/mol, Selleckchem) were dissolved in dimethyl sulfoxide-d_6_ (99.96 atom % D, Sigma-Aldrich).

### Purification of YaxAB nanopores and proteins

The *Yersinia enterocolitica* orthologues YaxA (Gene ID 4715532) and YaxB (Gene ID 4715533) were synthesized by Cosmogenetech (Daejeon, Republic of Korea). Recombinant YaxA and YaxB proteins were expressed in BL21(DE3) *Escherichia coli* cells grown at 20 °C in a 2×YT medium overnight, following induction with 0.5 mM IPTG^26^. For purification of YaxA and YaxB, cell pellets were resuspended in a lysis buffer (50 mM Tris-HCl pH 8.0, 300 mM NaCl, 1 mM PMSF, 1 µg/mL DNase I, and 0.2 mg/mL lysozyme) and sonicated. The clarified lysate was loaded onto a 5 mL Ni-NTA affinity column (Cytiva, Marlborough, MA, USA) equilibrated with Ni-binding buffer (50 mM Tris-HCl pH 8.0, 300 mM NaCl, and 10 mM imidazole) and eluted with a linear imidazole gradient from 10 mM to 1 M. Pooled fractions were dialyzed overnight at 4 °C using a dialysis buffer (20 mM HEPES pH 7.0 and 25 mM NaCl) in the presence of thrombin protease. Next, YaxB samples were further purified on a 5 mL HiTrap Q column (Cytiva, Marlborough, MA, USA) equilibrated in dialysis buffer, and the bound proteins were eluted with a linear salt gradient of 25 mM-1 M NaCl. Bcl-xL (PDB code: “1BXL”) and FKBP12 (PDB code: “2PPN”) were expressed in *Escherichia coli* BL21 (DE3) cells grown in LB media at 37 °C for 4 h and at 18 °C overnight, respectively. Cell pellets were resuspended and sonicated. The clarified lysate was purified on a 5 mL Ni-NTA affinity column (Cytiva, Marlborough, MA, USA). Peak fractions were further purified on a Superdex75 16/60 gel filtration column (Cytiva, Marlborough, MA, USA). MDM2 (PDB code: “2MPS”) was expressed in BL21 (DE3) *Escherichia coli* cells grown in LB medium at 20 °C overnight, following induction with 0.4 mM IPTG. The protein was initially purified by ammonium sulfate-induced precipitation^51,52^. Further purification was conducted using HiTrap Q- and SP-Separose columns (Cytiva, Marlborough, MA, USA) and a gel filtration column (HiLoad 16/600 Superdex 75 pg, Cytiva, Marlborough, MA, USA)^53^. The holo-transferrin was purchased from Sigma-Aldrich (St. Louis, MO, USA). The dimensions of the proteins were measured by executing the Draw_Protein_Dimensions.py script in Pymol^54^.

### Preparation of the detergent-treated YaxAB complex

Typically, equal amounts of YaxA and YaxB were combined and incubated at 25 °C for 30 min. Cymal-6 (1.5% w/v) was added and the mixture was incubated at 4 °C for 30 min, then injected onto a Superose 6 column running in the gel filtration buffer (25 mM HEPES pH 7.0, 150 mM NaCl, 0.05% w/v Cymal-6). To separate different oligomeric states of protein complexes, peak fractions were separated by 4-16 % gradient blue native polyacrylamide gel electrophoresis (BN-PAGE; Thermo Fisher Scientific Korea, Invitrogen^TM^, South Korea) without any other additives. The bands corresponding to YaxAB-*C*_8_, -*C*_9_, and -*C*_10_ pores were cut out from the gel, soaked in the gel filtration buffer, and then incubated at 4 °C overnight to extract the proteins. The supernatant containing oligomeric YaxAB was used for the following experiments.

### Circular dichroism (CD) spectroscopy

CD spectra of Bcl-xL, FKBP12, and holo-transferrin were recorded in the wavelength range of 200– 250 nm with a CD spectrophotometer (Jasco J-815, Jasco Corp., Tokyo, Japan) at 20 °C. The optical path length was 1 mm, and the scan speed was 100 nm/min. Five scanning acquisitions were accumulated and averaged to obtain the final spectrum. The results were expressed as mean residue ellipticity [Θ] (deg·cm^2^·dmol^−1^).

### Surface plasma resonance (SPR) experiments

Proteins were immobilized by NTA-his tag capture using a NiHC1000M sensor chip (XanTec bioanalytics GmbH, Duesseldorf, Germany). The running buffer was 10 mM HEPES pH 7.4, 150 mM NaCl, 50 μM EDTA, and 0.005% Tween 20. His-Bcl-xL (1.5 μg/mL) in the running buffer was injected at 10 μL/min for 90 sec across one spot. A concentration series of each compound was injected at a flow rate of 30 μL/min at 25 °C. The association time and dissociation time were 2 and 6 min, respectively. The buffer blanks were also injected periodically for double referencing. The surface was regenerated between binding cycles with a 3 min injection of 350 mM EDTA. Fresh protein was injected at 10 μL/min for 90 sec at the beginning of each binding cycle. All sensorgram data were processed by using double referencing. To obtain kinetic rate constants (*k*_a_ and *k*_d_), corrected response data were then fit to a one-to-one binding site model. The equilibrium dissociation constant (*K*_D_) was determined by *k*_d_/*k*_a_.

### Negative-stain electron microscopy (EM) and fitting model structure

BN-PAGE-extracted YaxAB-*C*_8_ pores were applied to glow-discharged, carbon-coated grids (carbon film 400 mesh Cu, Electron Microscopy Sciences, Hatfield, PA, USA) and stained using 0.8% (w/v) Uranyl Formate solution (Electron Microscopy Sciences, Hatfield, PA, USA). Images were acquired at 67,000-fold magnification on a TECNAI G2 Sprit TWIN transmission electron microscope (FEI Company, Hillsboro, OR, USA) operated at 120 kV using an Eagle 4 K High Sensitivity CCD detector (FEI company, Hillsboro, OR, USA). Automated particle selection and 2D classification were performed using CryoSPARC. The initial model of YaxAB-*C*_8_ for the negative stain 3D reconstruction was generated from three representative classes of 2D averages with *C*_8_ symmetry, and 5,000 particles were selected for 3D reconstruction. Based on a set of YaxAB heterodimers from the YaxAB-*C*_10_ model (PDB code: “6EL1”), we generated the full model structure of YaxAB-*C*_8_ through the rigid body fitting using Coot software^55^. The model was subjected to several rounds of geometry minimization using phenix.geometry minimization, including the secondary structure and NCS restraints.

### MD simulations

To characterize the YaxAB-*C*_8_ nanopore using the molecular dynamics (MD) simulation, we constructed a simulation setup with a YaxAB-*C*_8_ nanopore embedded in a lipid membrane. We prepared a lipid bilayer system that measured about 25 × 25 nm by duplicating a pre-equilibrated membrane taken from our previous study^56^. We placed the YaxAB-*C*_8_ nanopore structure in the lipid membrane such that the transmembrane domain of the nanopore was within the lipid membrane. Then, the lipid molecules in contact with the nanopore were removed using the VMD program^57^. We immersed the complex system of the lipid membrane and the nanopore in a solution of 1 M KCl. We performed all MD simulations using the GROMACS 2020.2 package^58^. We employed the CHARMM36m force field^59^ combined with the CHARMM-modified TIP3P model. To improve charge-charge interaction pairs, we applied the CUFIX corrections to the CHARMM36m force field set^60^. We performed all simulations under a constant surface tension–constant temperature (NPγT) ensemble; surface tension was zero (γ = 0)^61^ and the temperature was 303 K^62^. For the computation of van der Waals forces, we employed a 10 to 12 Å switching scheme. We computed the long-range electrostatic forces using the particle-mesh Ewald summation scheme^63^ with a 1.2 Å grid spacing and a 12 Å real-space cutoff. Covalent bonds to hydrogen in non-water and water molecules were constrained using the LINCS^64^ and SETTLE^65^ algorithms, respectively. We visualized the density-flux map using the method described in a previous report by Yoo and Aksimentiev^56^.

To measure the free energy landscape through the YaxAB-*C*_8_ nanopore in the presence of Bcl-xL protein under the applied voltage (0, 30, and 100 mV), we added a Bcl-xL protein at the distance of 10 nm from the membrane, $z$, to the equilibrated membrane-nanopore complex system. For a given voltage bias, we performed the Umbrella sampling MD simulations^66^ by applying a harmonic force to constrain $z$ at a fixed equilibrium distance, ranging from 2 nm to 13 nm with 1 nm spacing; note that the Bcl-xL protein freely moved in x and y directions. Note that the applied constraints enhance the sampling in the transition states (positions not in the energy minima) without affecting the overall shape of the free energy^66^. For each constraining $z$ position, we computed the mean force from a 70 ns simulation. By integrating the mean forces from $z=2$ to 13 nm, we obtained the free energy landscape. In all MD simulations, we determined the protonation states of histidine residues at pH 7. To quantify the effects of the voltage bias on the free energy landscape, we performed three sets of Umbrella sampling simulations under 0, 30, and 100 mV.

### Brownian dynamics simulations

To estimate the vibrational motion of Bcl-xL inside the nanopore, we performed the Brownian dynamics simulations along the nanopore channel ($z$) using the algorithm of Ermak and McCammon^67^. Specifically, we numerically solved the following Brownian equation Eq. 1.

1 $$z\left(t+\triangle t\right)=z\left(t\right)+\frac{D}{{k}_{B}T}F\left(z\right)\triangle t+S$$

where $z(t)$ is the position of Bcl-xL along the nanopore axis; $F(z)$, $D$, and $S$ are the thermodynamic force at $z$, the diffusion coefficient of proteins (10 nm^2^/µs), and a random number generated from a normal distribution having the width of $2{{{{{\rm{D}}}}}}\triangle {{{{{\rm{t}}}}}},\,$respectively. The force $F(z)$ was computed as a numerical derivative of the free energy landscape $\triangle G(z)$ from the MD simulation: $F\left(z\right)=-d\triangle G(z)/{dz}$. The integration time step (∆t) was 0.0001 µs.

### Single-channel recordings using the YaxAB-*C*_8_ nanopore

A planar lipid bilayer was formed on a Teflon film with a 100 μm aperture using a painting method^68^. Briefly, lipid powder was dissolved in chloroform and dried under a gentle stream of argon gas to remove the solvent. The dried lipid stock was then redissolved in n-decane to a final concentration of 3.0% (w/v). The lipid stock was prepainted on both sides of the aperture prepared in the Teflon film, which was then fixed in between two customized Teflon chambers and dried to remove the solvent. After drying, each chamber was filled with 0.8 mL solution containing 10 mM Tris-HCl pH 7.5, 1 mM EDTA, and 1 M KCl. Ag/AgCl electrodes were dipped into the *trans* and *cis* chambers of the setup to make electrical measurements. The ground electrode was connected to the *trans* side and the working electrode to the *cis* side. To form a single YaxAB nanopore in the lipid membrane, a thimbleful of purified YaxAB-*C*_8_ samples was added to the *cis* chamber. Nanopore current signals were acquired at the applied voltage of +100 mV (at the *cis* side). After insertion of the YaxAB nanopore, various proteins of 100-2000 nM (Bcl-xL, holo-transferrin, FKBP12, MDM2, and Bak-BH3) and small-molecule drugs (ABT-737, LCL-161, GDC-0152, and A-1331852) were added to the *cis* chamber. Electrical recordings were performed under 40–100 mV engaged bias voltages using a patch-clamp amplifier (Axopatch 200B, Molecular Devices Inc., Sunnyvale, CA, USA). Nanopore data were acquired using pClamp 11 software (Molecular Devices Inc., Sunnyvale, CA, USA). The current signal, recorded at sampling rates of 100 kHz, was filtered with a built-in 8-pole low-pass Bessel Filter at 10 kHz. All nanopore experiments were performed at 25 °C.

### Electric recording data analysis

All nanopore events were analyzed by using Clampfit 11.2 software. The current blockade was described as ΔI/I_o_, where ΔI and I_o_ are the analyte-specific current blockade and the open pore current, respectively. The mean value of the current blockade (ΔI/I_o_) and the dwell time (τ) were calculated based on the peak values in the histograms fitted to a Gaussian function and a single exponential decay function, respectively. For noise analysis, current traces were additionally filtered with a cutoff frequency of 100 or 1000 Hz. Current noise (I_N_) was derived from power spectral density (PSD) analysis based on a nanopore event signal. The I_N_ value was computed by taking the square root of the integral of the power spectrum, I_N_ = $\scriptstyle\sqrt{\mathop{\sum }\nolimits_{n=1}^{N}{P}_{i}f/N}$, where *P*_i_ is the power at each sampling point, and *f/N* is the value of the frequency bin width in Hz. The first one spectral bin of the periodogram was excluded when we performed root-mean-square (RMS) measurement because the values in the first bin of a periodogram can be unreliable due to discontinuities at the edges of the original time domain signal, giving rise to spectral leakage in the frequency domain. The I_N_ measurement was based on at least three independent nanopore measurements. Dissociation constant (*K*_D_) values of Bcl-xL/Bak-BH3, Bcl-xL/ABT-737, and Bcl-xL/A-1331852 interactions were determined through the I_N_ analysis of Bcl-xL (100 nM) with titrations of Bak-BH3, ABT-737, and A-1331852, respectively. The event frequency derived from each protein sample was calculated by counting the number of events at least three independent replicates. Based on the three independent nanopore measurements of each titration, we determined the bound fraction (*F*_c_, %) of the Bcl-xL/Bak-BH3 or Bcl-xL/ABT-737 or Bcl-xL/A-1331852 complex at each titration using the following equation: *F*_c_ = (*N*_comp_/*N*_total_) × 100, where *N*_comp_ and *N*_total_ are the number of events for complexes and the total number of events for the titrated binding partner (Bak-BH3, ABT-737 or A-1331852), respectively. The bound fraction was plotted against the concentration of the binding partner, and the data were fitted to one site-specific binding to provide standard curves for proteins and their ligands (Supplementary Fig. 17). At sufficiently high ligand concentration, the curve achieved the saturation point and reached the *B*_max_ value, and *K*_D_ was calculated using the Eq. 2.

2 $$Y=\,\frac{{B}_{\max }X}{{K}_{{{{{{\rm{D}}}}}}}+X}$$

where X is the concentration of the ligand, *B*_max_ is the maximum bound fraction in the same units as Y, and *K*_D_ is the dissociation constant. The parameters (*B*_max_ and *K*_D_) determined from fitting to one-site specific binding model showed the values within 95% confidence intervals (CI). The obtained *B*_max_ values of Bcl-xL/Bak-BH3, Bcl-xL/ABT-737, and Bcl-xL/A-1331852 complexes are 1.09 ± 0.06, 1.10 ± 0.06, and 1.11 ± 0.06, respectively. The *K*_D_ and *B*_max_ values determined in this study are consistent with 1:1 complex formation between Bcl-xL and Bak-BH3 peptide as previously reported^23^.

### Calculation of YaxAB pore open conductivity

The theoretical ionic currents were calculated based on constriction diameters derived from YaxAB models (Eq. 3)^27^.

3 $${R}_{{pore}}(\alpha )=\frac{4}{{\sigma }_{s}\pi }\cdot \frac{L}{{d}_{{in}}\cdot {d}_{{out}}}$$

*R*_pore_, pore resistance; *L*, nanopore channel length; *σ*_s_, electrolyte solution conductivity; *d*_in_, diameter of entry side; *d*_out_, diameter of exit side.

### Calculation of YaxAB pore ion selectivity

The ion selectivity (${P}_{{K}^{+}}/{P}_{{{Cl}}^{-}}$) of the YaxAB nanopore was calculated using the Goldman−Hodgkin−Katz equation (Eq. 4)^13^.

4 $$\frac{{P}_{{K}^{+}}}{{P}_{{{Cl}}^{-}}}=\frac{{\left[{\alpha }_{{{Cl}}^{-}}\right]}_{{cis}}-{\left[{\alpha }_{{{Cl}}^{-}}\right]}_{{trans}}{e}^{{V}_{r}F/{RT}}}{{\left[{\alpha }_{{K}^{+}}\right]}_{{cis}}{e}^{{V}_{r}F/{RT}}-{\left[{\alpha }_{{K}^{+}}\right]}_{{trans}}}$$

where ${[{\alpha }_{{K}^{+}/{{Cl}}^{-}}]}_{{cis}/{trans}}$ is the activity of the K^+^ or Cl^-^ ions in the *cis* or *trans* chambers, R is the ideal gas constant, T is the temperature, F is Faraday’s constant, and V_r_ is the reversal potential, which is measured using asymmetric salt condition (*cis*: *trans*, 0.5 M:2 M of KCl). First, a single YaxAB nanopore was inserted into a symmetric salt condition (800 μL, 1 M KCl, 10 mM Tris-HCl, 1 mM EDTA, pH 7.5 buffer in both chambers). After adjustment of offset and balancing electrodes, 400 μL of the 1 M KCl solution was discarded in both chambers. Then, 400 μL of 3 M KCl was added to the *trans* chamber, and the same volume of salt-free buffer was added into the *cis* chamber to induce a salt gradient. Ion activity was derived from the mean ion activity coefficients (0.573 for 2 M KCl, and 0.649 for 0.5 M KCl^69^), multiplied by the molar concentration of the given ion. The solution in both chambers was carefully mixed, and I-V curves were measured to find reversal potentials. The ground electrode was connected to the *trans* side and the working electrode to the *cis* side.

### Statistics and reproducibility

Electrical recordings were performed using a patch-clamp amplifier (Axopatch 200B, Molecular Devices Inc., Sunnyvale, CA, USA), and nanopore data were acquired using the pClamp 11 software (Molecular Devices Inc.). All analysis and visualizations were performed with GraphPAD Prism 9 and Origin 2020b, Pymol 2.5.0, VMD 1.9.4, Coot 0.8.9.2, HOLE 2.2.005, cryoSPARC v3.2, and Phenix 1.18.2. For electrical signal analysis, we used Clampfit 11.2. Each specific data point was derived from at least three independent experimental replicates at the same conditions. The sample size was determined by the reproducibility of the experimental observation, such as the electrical signal pattern or the convergence of the cumulative mean of independent samples. All signal traces exhibiting the known behavior of the YaxAB pore were used indiscriminately. Randomization was not applicable for this work, as the object in this study was a specific protein under a specific experimental condition. Blinding was not applicable, as the object in this study was a specific protein.

### Reporting summary

Further information on research design is available in the Nature Portfolio Reporting Summary linked to this article.

## Supplementary information

Supplementary information

Reporting Summary

## Data Availability

The source data underlying Figs. 1a, 1c, 1g, 2h, 3b, 3c, 3e, 3f, 4b, 4c, 5c, 5d, 5f, 5g and Supplementary Figs. 1, 2, 3–9, 11–12, 17–24 and Supplementary Table 1, 2, and 3 are provided in the Source data file. PDB entries “6EL1”, “1BXL”, “2PPN”, and “2MPS” were downloaded from the Protein Data Bank and used in this article for molecular visualizations. Raw micrographs of negative-staining electron microscopy (EM) for pores generated in this study have been deposited in the Zenodo (10.5281/zenodo.7619077). Source data are provided with this paper.

## Source data

Source data

## References

[1] Jing X, Ji P, Schrieber SJ, Fletcher EP, Sahajwalla C (2020). Update on Therapeutic Protein-Drug Interaction: Information in Labeling. Clin. Pharmacokinet..

[2] Iorio F (2010). Discovery of drug mode of action and drug repositioning from transcriptional responses. Proc. Natl Acad. Sci..

[3] Frostell A, Vinterback L, Sjobom H (2013). Protein-Ligand Interactions Using SPR Systems. Methods Mol. Biol..

[4] Damian L (2013). Isothermal titration calorimetry for studying protein-ligand interactions. Methods Mol. Biol..

[5] Rossi AM, Taylor CW (2011). Analysis of protein-ligand interactions by fluorescence polarization. Nat. Protoc..

[6] Divakaran A, Kirberger SE, Pomerantz WCK (2019). SAR by (Protein-Observed)^19^F NMR. Acc. Chem. Res..

[7] Alfaro JA (2021). The emerging landscape of single-molecule protein sequencing technologies. Nat. Methods..

[8] Mayer SF, Cao C, Dal Peraro M (2022). Biological nanopores for single-molecule sensing. Iscience.

[9] Varongchayakul N, Song J, Meller A, Grinstaff MW (2018). Single-molecule protein sensing in a nanopore: a tutorial. Chem. Soc. Rev..

[10] Wang S, Zhao Z, Haque F, Guo P (2018). Engineering of protein nanopores for sequencing, chemical or protein sensing and disease diagnosis. Curr. Opin. Biotechnol..

[11] Thakur AK, Movileanu L (2019). Real-time measurement of protein-protein interactions at single-molecule resolution using a biological nanopore. Nat. Biotechnol..

[12] Fahie M, Chisholm C, Chen M (2015). Resolved single-molecule detection of individual species within a mixture of anti-biotin antibodies using an engineered monomeric nanopore. ACS Nano..

[13] Huang G (2020). Electro-Osmotic Vortices Promote the Capture of Folded Proteins by PlyAB Nanopores. Nano Lett..

[14] Schmid S, Stommer P, Dietz H, Dekker C (2021). Nanopore electro-osmotic trap for the label-free study of single proteins and their conformations. Nat. Nanotechnol..

[15] Kwak DK (2016). Probing the Small-Molecule Inhibition of an Anticancer Therapeutic Protein-Protein Interaction Using a Solid-State Nanopore. Angew. Chem., Int. Ed. Engl..

[16] Zernia S, van der Heide NJ, Galenkamp NS, Gouridis G, Maglia G (2020). Current Blockades of Proteins inside Nanopores for Real-Time Metabolome Analysis. ACS Nano..

[17] Li X, Lee KH, Shorkey S, Chen J, Chen M (2020). Different Anomeric Sugar Bound States of Maltose Binding Protein Resolved by a Cytolysin A Nanopore Tweezer. ACS Nano..

[18] Galenkamp NS, Soskine M, Hermans J, Wloka C, Maglia G (2018). Direct electrical quantification of glucose and asparagine from bodily fluids using nanopores. Nat. Commun..

[19] Arkin MR, Wells JA (2004). Small-molecule inhibitors of protein-protein interactions: progressing towards the dream. Nat. Rev. Drug Discov..

[20] Mabonga L, Kappo AP (2019). Protein-protein interaction modulators: advances, successes and remaining challenges. Biophysical Rev..

[21] Vogler M, Dinsdale D, Dyer MJ, Cohen GM (2009). Bcl-2 inhibitors: small molecules with a big impact on cancer therapy. Cell Death Differ..

[22] Chittenden T (1995). A conserved domain in Bak, distinct from BH1 and BH2, mediates cell death and protein binding functions. EMBO J..

[23] Sattler M (1997). Structure of Bcl-xL-Bak peptide complex: recognition between regulators of apoptosis. Science.

[24] Kline MP (2007). ABT-737, an inhibitor of Bcl-2 family proteins, is a potent inducer of apoptosis in multiple myeloma cells. Leukemia.

[25] Wagner NJ, Lin CP, Borst LB, Miller VL (2013). YaxAB, a Yersinia enterocolitica pore-forming toxin regulated by RovA. Infect. Immun..

[26] Brauning B (2018). Structure and mechanism of the two-component alpha-helical pore-forming toxin YaxAB. Nat. Commun..

[27] Edel, J. B. & Albrecht, T. Engineered Nanopores for Bioanalytical Applications. (William Andrew, 2013).

[28] Piguet F (2014). Electroosmosis through alpha-Hemolysin That Depends on Alkali Cation Type. J. Phys. Chem. Lett..

[29] Davenport M (2012). The role of pore geometry in single nanoparticle detection. ACS Nano..

[30] Liu Y (2022). Machine Learning Assisted Simultaneous Structural Profiling of Differently Charged Proteins in a Mycobacterium smegmatis Porin A (MspA) Electroosmotic Trap. J. Am. Chem. Soc..

[31] Asandei A (2016). Electroosmotic Trap Against the Electrophoretic Force Near a Protein Nanopore Reveals Peptide Dynamics During Capture and Translocation. ACS Appl. Mater. Interfaces..

[32] Huang G (2022). PlyAB Nanopores Detect Single Amino Acid Differences in Folded Haemoglobin from Blood. *Angew. Chem*. Int. Ed. Engl..

[33] Helmerhorst E, Chandler DJ, Nussio M, Mamotte CD (2012). Real-time and Label-free Bio-sensing of Molecular Interactions by Surface Plasmon Resonance: A Laboratory Medicine Perspective. Clin. Biochem. Rev..

[34] Wang L (2020). Discovery of A-1331852, a First-in-Class, Potent, and Orally-Bioavailable BCL-XL Inhibitor. ACS Med. Chem. Lett..

[35] Hamilton GS, Steiner JP (1998). Immunophilins: beyond immunosuppression. J. Med. Chem..

[36] Zhao YY, Mandal R, Li XF (2005). Intact human holo-transferrin interaction with oxaliplatin. Rapid Commun. Mass Spectrom..

[37] Primikyri A (2014). Direct binding of Bcl-2 family proteins by quercetin triggers its pro-apoptotic activity. ACS Chem. Biol..

[38] Li F, Fahie MA, Gilliam KM, Pham R, Chen M (2022). Mapping the conformational energy landscape of Abl kinase using ClyA nanopore tweezers. Nat. Commun..

[39] Kwak DK, Kim JS, Lee MK, Ryu KS, Chi SW (2020). Probing the Neuraminidase Activity of Influenza Virus Using a Cytolysin A Protein Nanopore. Anal. Chem..

[40] Biesemans A, Soskine M, Maglia G (2015). A Protein Rotaxane Controls the Translocation of Proteins Across a ClyA Nanopore. Nano Lett..

[41] Lu B (2018). Protein Motion and Configurations in a Form-Fitting Nanopore: Avidin in ClyA. Biophys. J..

[42] Rosen CB, Rodriguez-Larrea D, Bayley H (2014). Single-molecule site-specific detection of protein phosphorylation with a nanopore. Nat. Biotechnol..

[43] Liu Y (2021). Allosteric Switching of Calmodulin in a Mycobacterium smegmatis porin A (MspA) Nanopore-Trap. *Angew. Chem*. Int. Ed. Engl..

[44] Sun J, Thakur AK, Movileanu L (2020). Protein Ligand-Induced Amplification in the 1/f Noise of a Protein-Selective Nanopore. Langmuir.

[45] Yusko EC (2017). Real-time shape approximation and fingerprinting of single proteins using a nanopore. Nat. Nanotechnol..

[46] Waduge P (2017). Nanopore-Based Measurements of Protein Size, Fluctuations, and Conformational Changes. ACS Nano..

[47] Fragasso A, Schmid S, Dekker C (2020). Comparing Current Noise in Biological and Solid-State Nanopores. ACS Nano..

[48] Sutton MA (2019). Radiation Tolerance of Nanopore Sequencing Technology for Life Detection on Mars and Europa. Sci. Rep..

[49] Leggett RM, Clark MD (2017). A world of opportunities with nanopore sequencing. J. Exp. Bot..

[50] Obergrussberger A (2022). The suitability of high throughput automated patch clamp for physiological applications. J. Physiol..

[51] Yoon MK (2018). Cytoplasmic pro-apoptotic function of the tumor suppressor p73 is mediated through a modified mode of recognition of the anti-apoptotic regulator Bcl-XL. J. Biol. Chem..

[52] Oh S, Lee MK, Chi SW (2021). Single-molecule analysis of interaction between p53TAD and MDM2 using aerolysin nanopores. Chem. Sci..

[53] Szep S, Park S, Boder ET, Van Duyne GD, Saven JG (2009). Structural coupling between FKBP12 and buried water. Proteins: Struct. Funct. Genet..

[54] Guardado-Calvo, P. Python script to calculate and draw a minimal bounding box for a given protein. (2016).

[55] Emsley P, Lohkamp B, Scott WG, Cowtan K (2010). Features and development of Coot. Acta Crystallogr. D..

[56] Yoo J, Aksimentiev A (2015). Molecular Dynamics of Membrane-Spanning DNA Channels: Conductance Mechanism, Electro-Osmotic Transport, and Mechanical Gating. J. Phys. Chem. Lett..

[57] Humphrey W, Dalke A, Schulten K (1996). VMD: Visual molecular dynamics. J. Mol. Graph..

[58] Abraham MJ (2015). GROMACS: High performance molecular simulations through multi-level parallelism from laptops to supercomputers. SoftwareX.

[59] Huang J (2017). CHARMM36m: an improved force field for folded and intrinsically disordered proteins. Nat. Methods..

[60] Yoo J, Aksimentiev A (2018). New tricks for old dogs: improving the accuracy of biomolecular force fields by pair-specific corrections to non-bonded interactions. Phys. Chem. Chem. Phys..

[61] Parrinello M, Rahman A (1981). Polymorphic transitions in single crystals: A new molecular dynamics method. J. Appl. Phys..

[62] Nosé S, Klein ML (1983). Constant pressure molecular dynamics for molecular systems. Mol. Phys..

[63] Darden T, York D, Pedersen L (1993). Particle mesh Ewald: An N⋅log(N) method for Ewald sums in large systems. J. Chem. Phys..

[64] Hess B, Bekker H, Berendsen HJC, Fraaije JGEM (1997). LINCS: A linear constraint solver for molecular simulations. J. Comput. Chem..

[65] Miyamoto S, Kollman PA (1992). Settle: An analytical version of the SHAKE and RATTLE algorithm for rigid water models. J. Comput. Chem..

[66] Kumar S, Rosenberg JM, Bouzida D, Swendsen RH, Kollman PA (1992). The weighted histogram analysis method for free‐energy calculations on biomolecules. I. The method. J. Comput. Chem..

[67] Ermak DL, McCammon JA (1978). Brownian dynamics with hydrodynamic interactions. J. Chem. Phys..

[68] Jeong K-B (2019). Alpha-Hederin nanopore for single nucleotide discrimination. ACS Nano..

[69] Khoshkbarchi MK, Vera JH (1996). Measurement and correlation of ion activity in aqueous single electrolyte solutions. AIChE J..

