# Supplementary material for: Single-molecule fingerprinting of protein-drug interaction using a funneled biological nanopore
Source: Nat Commun. 2023 Apr 4;14:1461. doi: 10.1038/s41467-023-37098-4 (PMC10073129; doi:10.1038/s41467-023-37098-4)
Supplement: Supplementary file 1 — Supplementary information [file 41467_2023_37098_MOESM1_ESM.pdf]

Supplementary Information

Single-molecule fingerprinting of protein-drug interaction using a funneled biological nanopore

Ki-Baek Jeong[1,2,†], Minju Ryu[1,3,†], Jin-Sik Kim[1,2,†], Minsoo Kim[4], Jejoong Yoo[4], Minji Chung[1], Sohee Oh[1], Gyunghee Jo[5], Seong-Gyu Lee[5], Ho Min Kim[5,6], Mi-Kyung Lee[1,2,3,*], Seung-Wook Chi[1,3,7,*]

[1]Disease Target Structure Research Center, Division of Biomedical Research, Korea Research Institute of Bioscience and Biotechnology (KRIBB), Daejeon 34141, Republic of Korea

[2]Critical Diseases Diagnostics Convergence Research Center, KRIBB, Daejeon 34141, Republic of Korea

[3]Department of Proteome Structural Biology, KRIBB School of Bioscience, University of Science and Technology, Daejeon 34113, Republic of Korea

[4]Department of Physics, Sungkyunkwan University, Suwon, Gyeonggi 16419, Republic of Korea

[5]Center for Biomolecular and Cellular Structure, Institute for Basic Science (IBS), Daejeon 34126, Republic of Korea

[6]Graduate School of Medical Science and Engineering, Korea Advanced Institute of Science and Technology (KAIST), Daejeon 34141, Republic of Korea

[7]School of Pharmacy, Sungkyunkwan University, Suwon, Gyeonggi 16419, Republic of Korea

Corresponding authors: miki@kribb.re.kr, swchi@kribb.re.kr

† These authors contributed equally.

Contents

Supplementary Fig. 1. Purification of YaxAB pores. 4

Supplementary Fig. 2. Negative-staining electron microscopy (EM) image of YaxAB pore and the structure of YaxAB-C_8. 5

Supplementary Fig. 3. Open conductance distributions of YaxAB nanopores..6

Supplementary Fig. 4. Electrophysiological characterization of YaxAB nanopores..7

Supplementary Fig. 5. The circular dichroism (CD) analysis of protein analytes..8

Supplementary Fig. 6. Measurement of dwell times of Bcl-xL using YaxAB nanopores at different voltages. 9

Supplementary Fig. 7. Current blockade analysis for Bcl-xL using YaxAB nanopores at different voltages..10

Supplementary Fig. 8. The capture rate and trapping event frequency of wild-type and mutant Bcl-xL measured using YaxAB nanopores at different voltages..11

Supplementary Fig. 9. The Brownian dynamics simulation used to model Bcl-xL movement within a YaxAB nanopore..12

Supplementary Fig. 10. Distinctive current signal patterns of Bcl-xL and its mutants measured by YaxAB nanopores at different voltages..13

Supplementary Fig. 11. Current blockade analysis of Bcl-xL/Bak-BH3 complex using YaxAB nanopores at different voltages..14

Supplementary Fig. 12. Current blockade-based analysis of Bcl-xL/Bak-BH3 interaction inhibition by ABT-737 using YaxAB nanopores..15

Supplementary Fig. 13. Orientation and strength of dipole moment for free Bcl-xL and three Bcl-xL complexes (Bcl-xL/Bak-BH3, Bcl-xL/ABT-737, and Bcl-xL/A-1331852)..............16

Supplementary Fig. 14. Current noise (I_N)-based analysis of nanopore events upon titration of Bcl-xL with Bak-BH3..17

Supplementary Fig. 15. Current noise (I_N)-based analysis of nanopore events upon titration of Bcl-xL with ABT-737..18

Supplementary Fig. 16. Current noise (I_N)-based analysis of nanopore events upon titration of Bcl-xL with A-1331852..19

Supplementary Fig. 17. Current noise (I_N) analysis-based determination of Bcl-xL binding affinity for Bak-BH3 peptide, ABT-737, and A-1331852 using YaxAB nanopores.................20

Supplementary Fig. 18. SPR analysis for interaction between Bcl-xL and small-molecule drugs..21

Supplementary Fig. 19. YaxAB nanopore measurements with non-specific small-molecule ligands..22

Supplementary Fig. 20. Detection of FKBP12/FK506 interaction using YaxAB nanopores....23

Supplementary Fig. 21. Detection of holo-transferrin/oxaliplatin interaction using YaxAB nanopores..24

Supplementary Fig. 22. Current blockade and noise analyses of Bcl-xL/Quercetin interactions using YaxAB nanopores..25

Supplementary Fig. 23. Current blockade and noise analyses of Bcl-xL/Bax-BH3 interactions using YaxAB nanopores..26

Supplementary Fig. 24. YaxAB nanopore measurements in the presence of DMSO.............27

Supplementary Table 1. Current-voltage (I-V) curves for YaxAB nanopores in symmetric salt conditions at pH 7.5...28

Supplementary Table 2. Current-voltage (I-V) curves for YaxAB nanopores in asymmetric salt conditions at pH 7.5...29

Supplementary Table 3. Ion selectivity of YaxAB nanopores..30

Supplementary Table 4. Molecular properties of Bcl-xL and its complexes.............................31

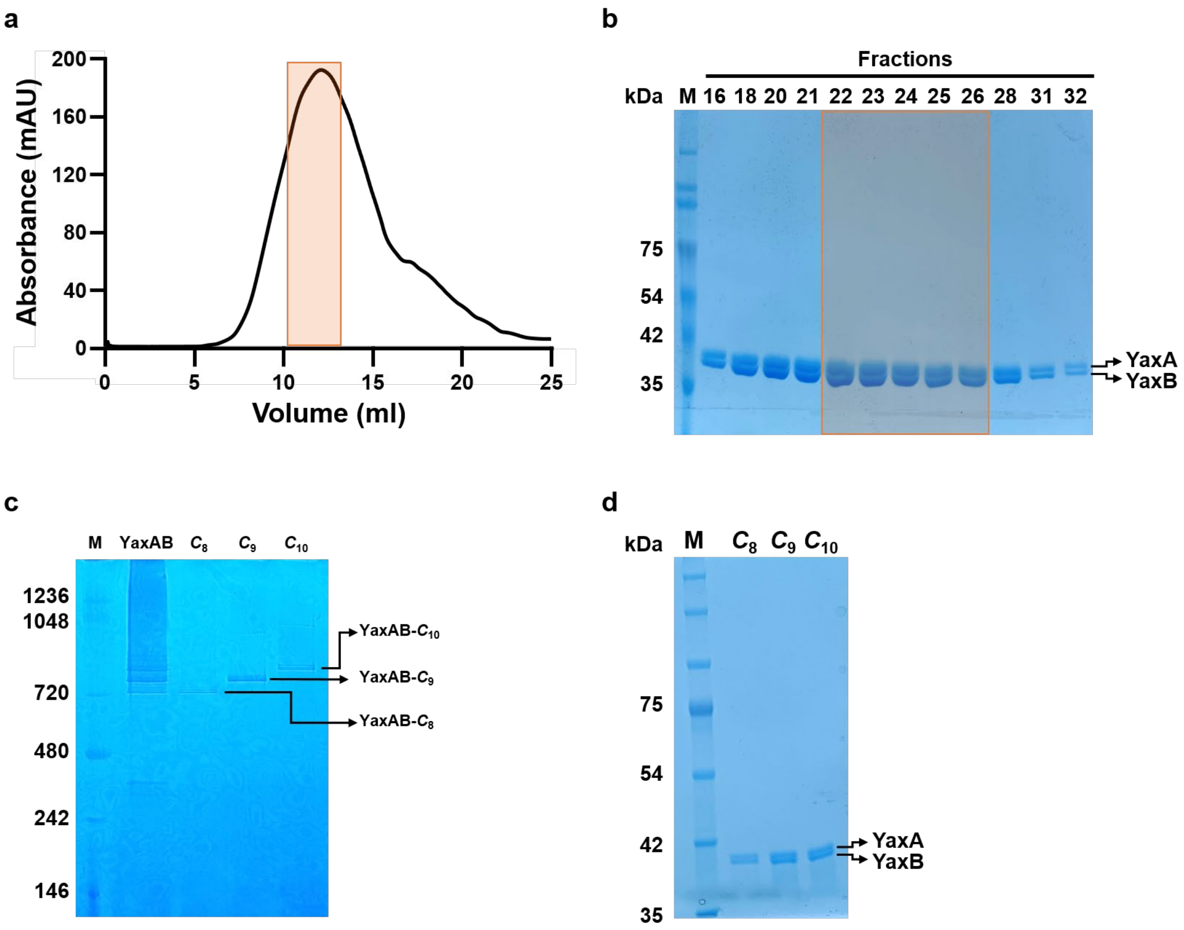

Supplementary Fig. 1: Purification of YaxAB pores. a-d. The chromatogram of size exclusion chromatography using the Superose 6 column (**a**) and SDS-PAGE gel image of purified YaxAB complex (**b**). (**c**) The native gel image of three YaxAB oligomeric states, YaxAB-C_8 (16-mer), YaxAB-C_9 (18-mer), and YaxAB-C_{10} (20-mer), separated via gel extraction. (**d**) The SDS-PAGE gel image of the three YaxAB oligomeric states (YaxAB-C_8, YaxAB-C_9, and YaxAB-C_{10}) extracted from gel. All gel images include a size marker (lane M). The representative PAGE result is shown (n = 3 independent replicates). Source data are provided as a Source Data file.

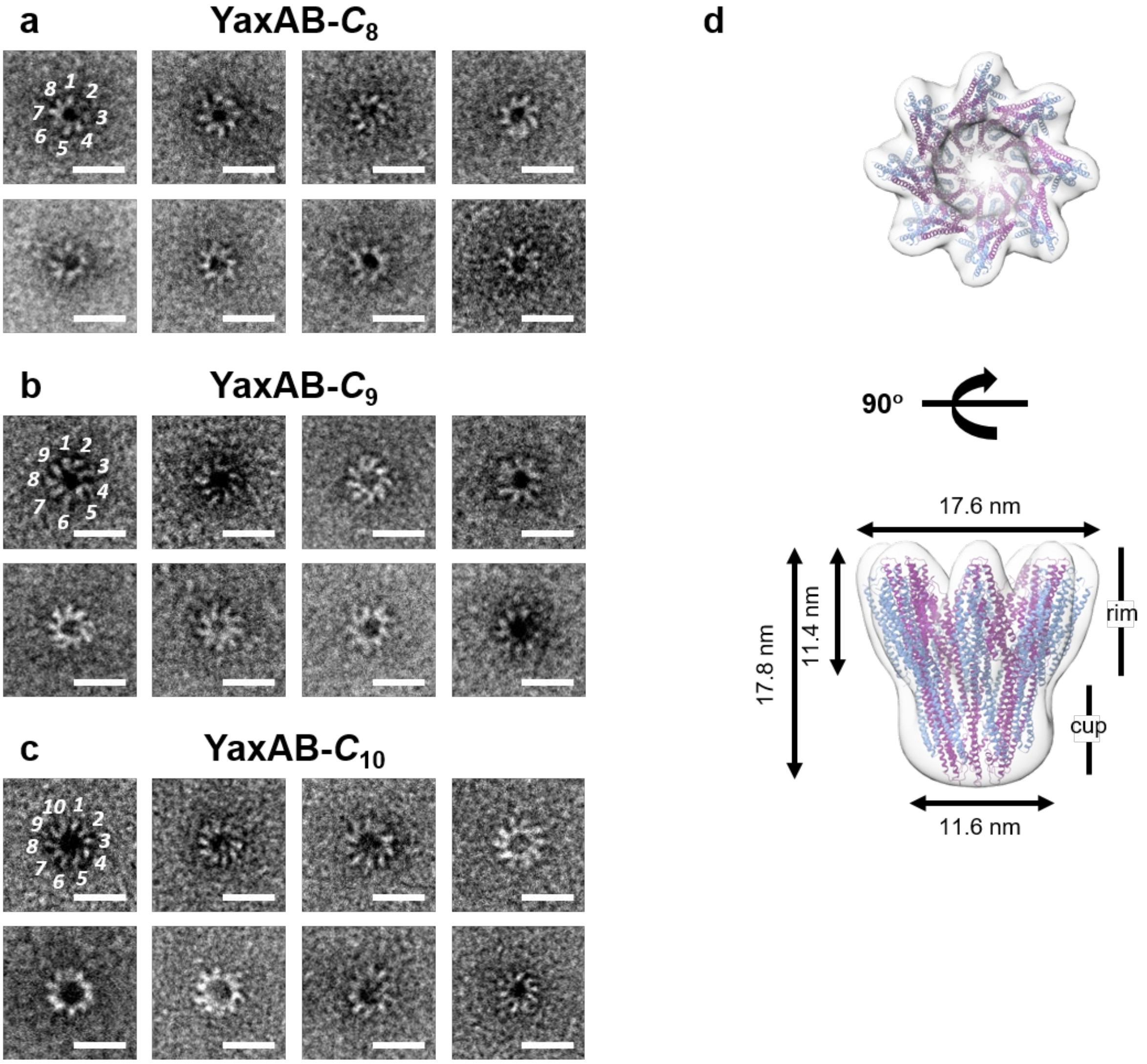

Supplementary Fig. 2: Negative-staining electron microscopy (EM) image of YaxAB pore and the structure of YaxAB-C_8. a-c. Top view of YaxAB-C_8 (**a**), YaxAB-C_9 (**b**), and YaxAB-C_{10} (**c**) pores cropped from raw micrographs of EM. The representative microscopy image is presented (n = 2 independent replicates). Source data are provided as a Source Data file. **d.** 3D volume and structural model of YaxAB-C_8 highlighting views along the C_8 symmetry axis and side views of the YaxAB-C_8 pore. YaxA and YaxB are colored blue and purple, respectively. Scale bar = 25 nm.

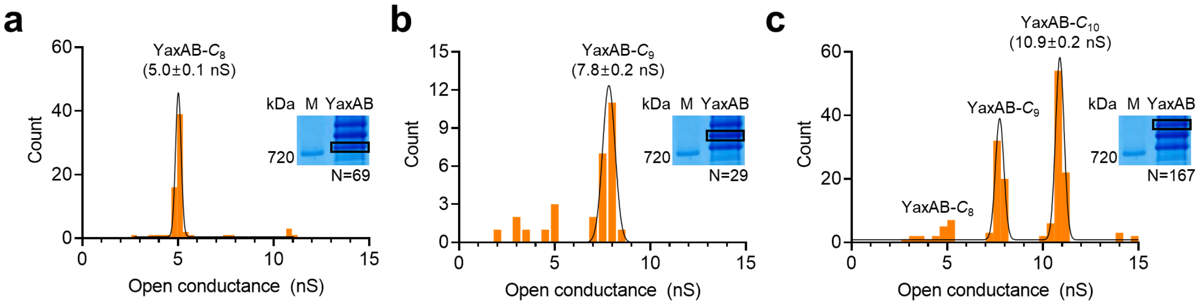

Supplementary Fig. 3: Open conductance distributions of YaxAB nanopores. a-c. Three types of YaxAB pore protein were collected from three separate bands on the blue native gel (4-16 %). Each conductance histogram was derived from nanopore experiments using YaxAB-C_8 (**a**), YaxAB-C_9 (**b**), or YaxAB-C_{10} (**c**) (black box). Conductance of each pore was recorded at 100 mV (*cis* side) in 1 M KCl at pH 7.5. In total, 265 nanopores were tested for pore insertion. All gel images include a size marker (lane M).

a

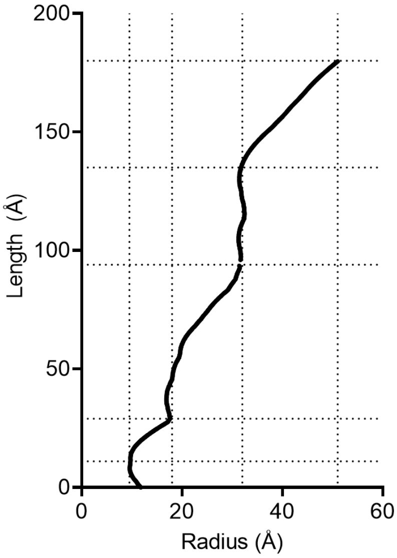

b

	Experimental (pA)	Theoretical (pA)
YaxAB-C_8	506.06 ± 6.35	542.58
YaxAB-C_9	776.04 ± 17.37	823.46
YaxAB-C_{10}	1086.52 ± 16.50	1173.88

Supplementary Fig. 4: Electrophysiological characterization of YaxAB nanopores. a. Radius and length of the YaxAB-C_8 nanopore were estimated using the program HOLE. **b.** Comparison between experimental and theoretical ionic currents of each pore. At an applied voltage of 100 mV, experimental and theoretical ionic currents were well-matched. The theoretical ionic currents were calculated from the inverse of the total resistance (R_{pore}) for funnel geometry of each pore, derived from YaxAB models (Equation 3, Methods section).

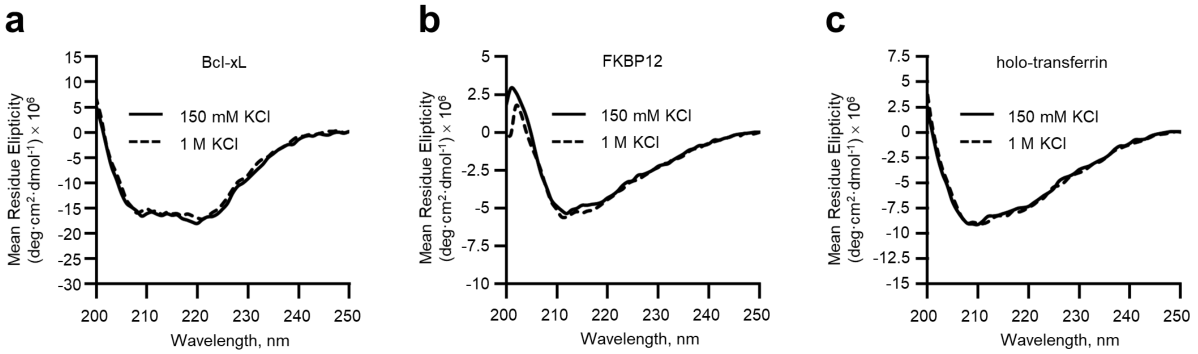

Supplementary Fig. 5. The circular dichroism (CD) analysis of protein analytes. a-c. The CD spectra of Bcl-xL (**a**), FKBP12 (**b**), and holo-transferrin (**c**) in the nanopore measurement buffer containing 150 mM and 1 M KCl.

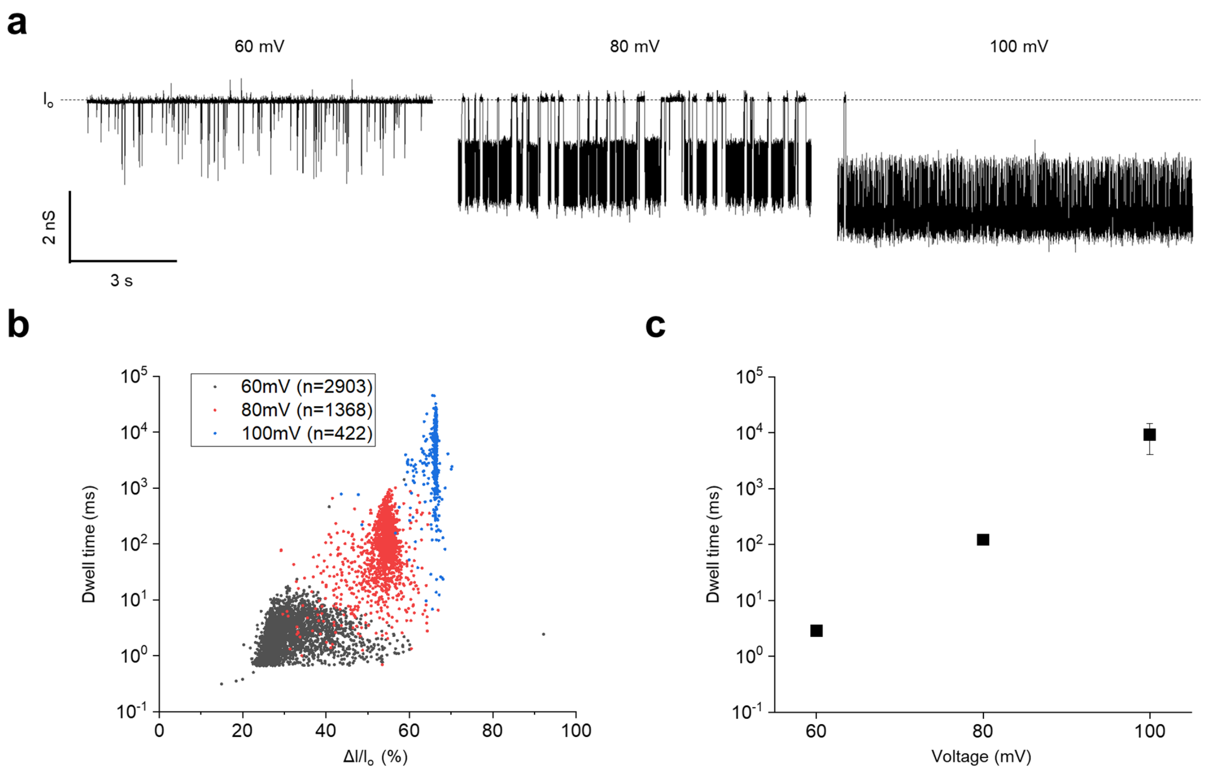

Supplementary Fig. 6: Measurement of dwell times of Bcl-xL using YaxAB nanopores at different voltages. a. Representative current traces of Bcl-xL at various applied voltages (60, 80, and 100 mV). **b.** The scatter plot of dwell time *vs.* relative current blockades ($\Delta I/I_o$) of nanopore events obtained at 60 mV (black), 80 mV (red), and 100 mV (blue). **c.** The voltage-dependence on dwell times of nanopore events obtained from Bcl-xL. The mean values of dwell times at 60, 80 and 100 mV are 2.85 ± 0.30 ms, 120.93 ± 12.45 ms, and 9292.96 ± 5200.98 ms, respectively. Data are presented as mean ± SD, n = 3 independent replicates for each engaged voltage. Source data are provided as a Source Data file.

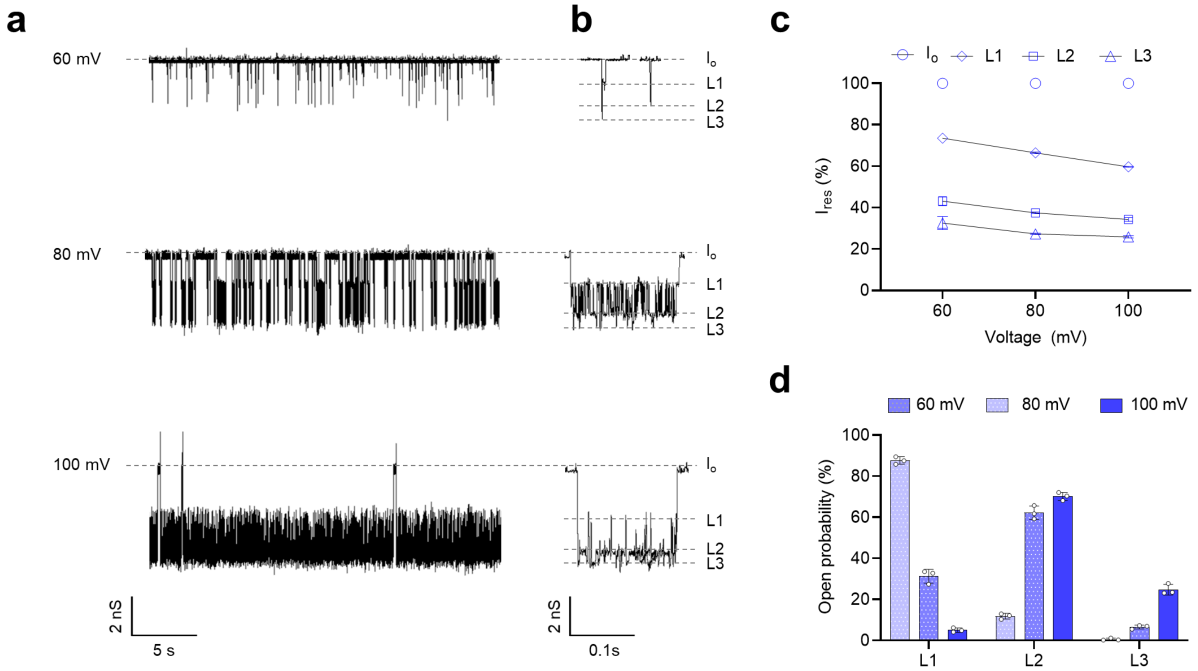

Supplementary Fig. 7: Current blockade analysis of Bcl-xL using YaxAB nanopores at different voltages. a. Representative current traces of Bcl-xL trapped by a YaxAB nanopore with engaged voltages (60, 80, and 100 mV) and a 1 kHz filter. I_o indicates open current of the nanopore, and each ionic current levels are L1, L2, and L3, respectively. Residual current (I_{res}) gradually decreased with voltage increase until 100 mV. **b.** Representative nanopore events exhibiting vibration signals. **c.** Residual current of Bcl-xL trapped by YaxAB nanopore decreased with voltage increment (60 to 100 mV). Data are presented as mean ± SD, n = 3 independent replicates for voltages (60, 80, and 100 mV). The ratio of residual current of L1 to L3 levels at different voltage biases; L1 (73.54 ± 1.08%), L2 (43.14 ± 2.19%), and L3 (32.54 ± 3.14%) at 60 mV; L1 (66.43 ± 0.36%), L2 (37.44 ± 0.42%), and L3 (27.33 ± 0.28%) at 80 mV; L1 (59.72 ± 0.17%), L2 (34.26 ± 0.87%), and L3 (25.85 ± 0.52%) at 100 mV. Source data are provided as a Source Data file. **d.** Open probability of each peak level in Bcl-xL as a function of engaged voltages. Data are presented as mean ± SD, n = 3 independent replicates for each engaged voltage. The open probability of L1 to L3 current levels at different voltage biases: L1 (87.62 ± 1.82%), L2 (11.82 ± 1.37%), and L3 (0.57 ± 0.54%) at 60 mV; L1 (31.25 ± 3.38%), L2 (62.18 ± 3.29%), and L3 (6.58 ± 0.92%) at 80 mV; L1 (5.04 ± 1.05%), L2 (70.19 ± 1.95%), and L3 (24.76 ± 2.6%) at 100 mV. Source data are provided as a Source Data file.

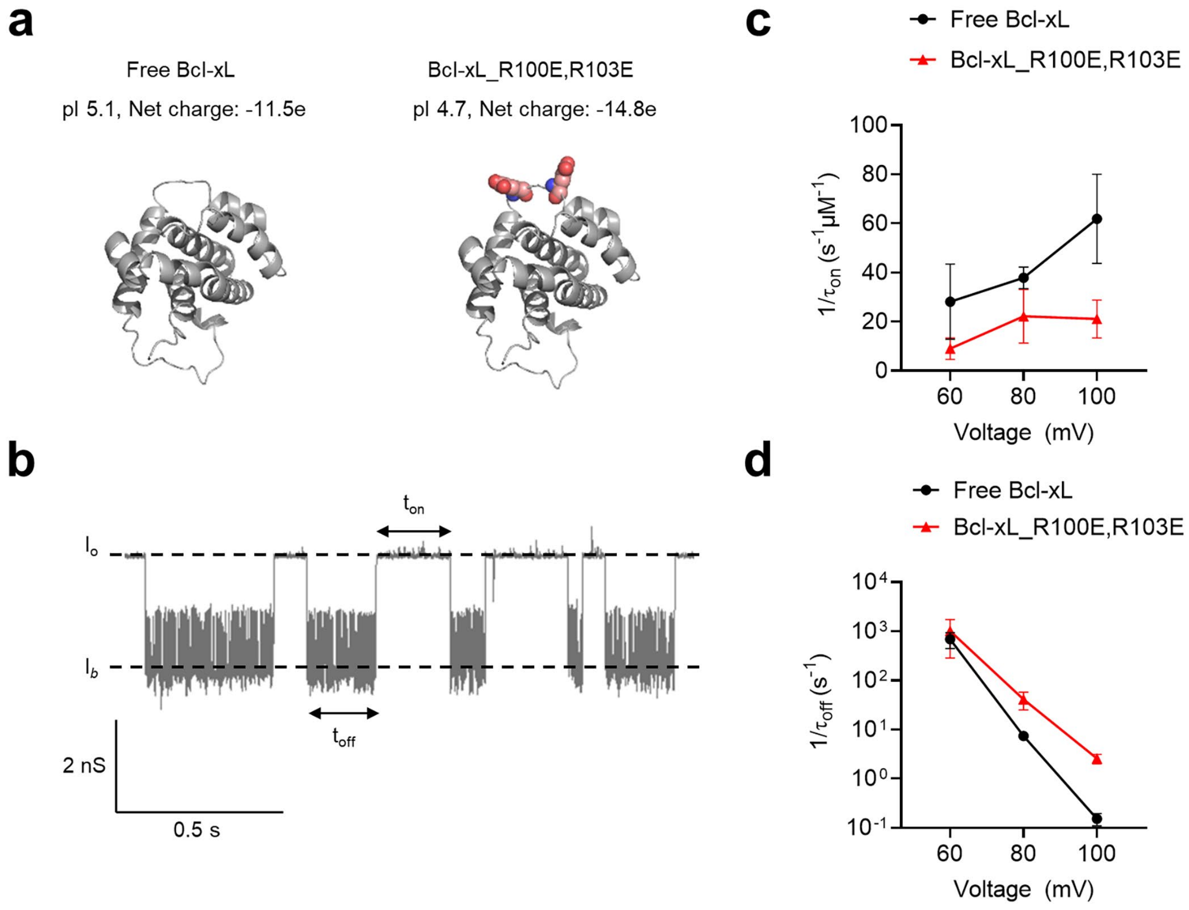

Supplementary Fig. 8: The capture rate and trapping event frequency of wild-type and mutant Bcl-xL measured by YaxAB nanopores at different voltages. a. Net-charge properties of wild-type and mutant Bcl-xL (Bcl-xL_R100E,R103E)**.** The mutation sites are indicated as spheres (red and blue) in Bcl-xL structure (gray). **b.** The representative current trace of wild-type Bcl-xL at 80 mV with the definition of event parameter. The interevent interval (t_{on}) and the dwell time (t_{off}) are defined as marked on the current trace. The mean time constants (τ_{on} and τ_{off}) were derived by fitting exponential decay with histograms of t_{on} and t_{off} at the various applied voltage (60, 80, and 100 mV), respectively. **c.** Plots of capture rate ($1/\tau_{on}$) *vs.* applied voltage of wild-type Bcl-xL (black circle) and mutant Bcl-xL (red triangle). Data are presented as mean ± SD, n = 3 independent replicates for wild-type Bcl-xL at 60 and 80 mV, n = 2 independent replicates for wild-type at 100 mV. The value of $1/\tau_{on}$: free Bcl-xL, 28.14 ± 15.31, 37.85 ± 4.38, 61.84 ± 18.16 $s^{-1}\mu M^{-1}$; the mutant Bcl-xL, 9.01 ± 4.40, 22.19 ± 10.92, 21.10 ± 7.70 $s^{-1}\mu M^{-1}$ at different voltage biases (60, 80, and 100 mV). Source data are provided as a Source Data file. **d.** Plots of $1/\tau_{off}$ *vs.* applied voltage. Data are presented as mean ± SD, n = 3 independent replicates. The value of $1/\tau_{off}$: free Bcl-xL, 691.47 ± 248.61, 7.38 ± 0.85, 0.15 ± 0.04 s^{-1}; the mutant Bcl-xL, 1013.97 ± 729.04, 41.15 ± 16.27, 2.57 ± 0.56 s^{-1} at different voltage biases (60, 80, and 100 mV). Source data are provided as a Source Data file.

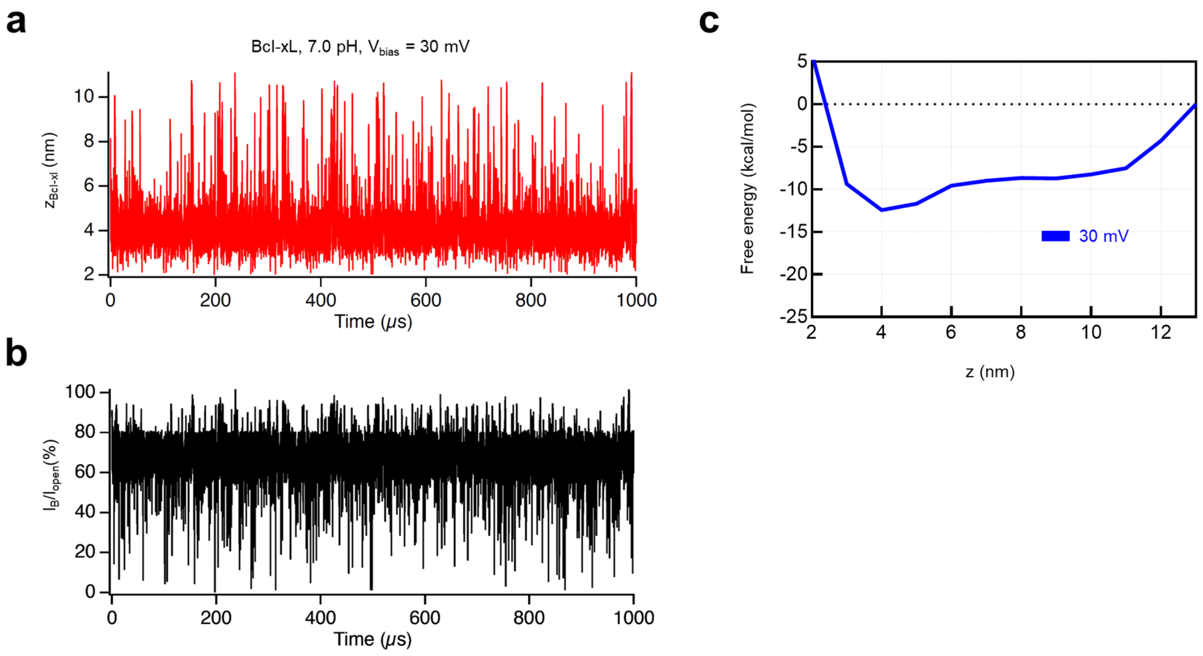

Supplementary Fig. 9: The Brownian dynamics simulation used to model Bcl-xL movement within a YaxAB nanopore. The position (**a**) and relative current blockade (**b**) of Bcl-xL were calculated by Brownian dynamics simulations performed at pH 7.0 at 30 mV. The condition was optimized to prove the presence of two levels of Bcl-xL in a YaxAB nanopore. (**c**) Free energy landscapes of Bcl-xL within a YaxAB nanopore at 30 mV.

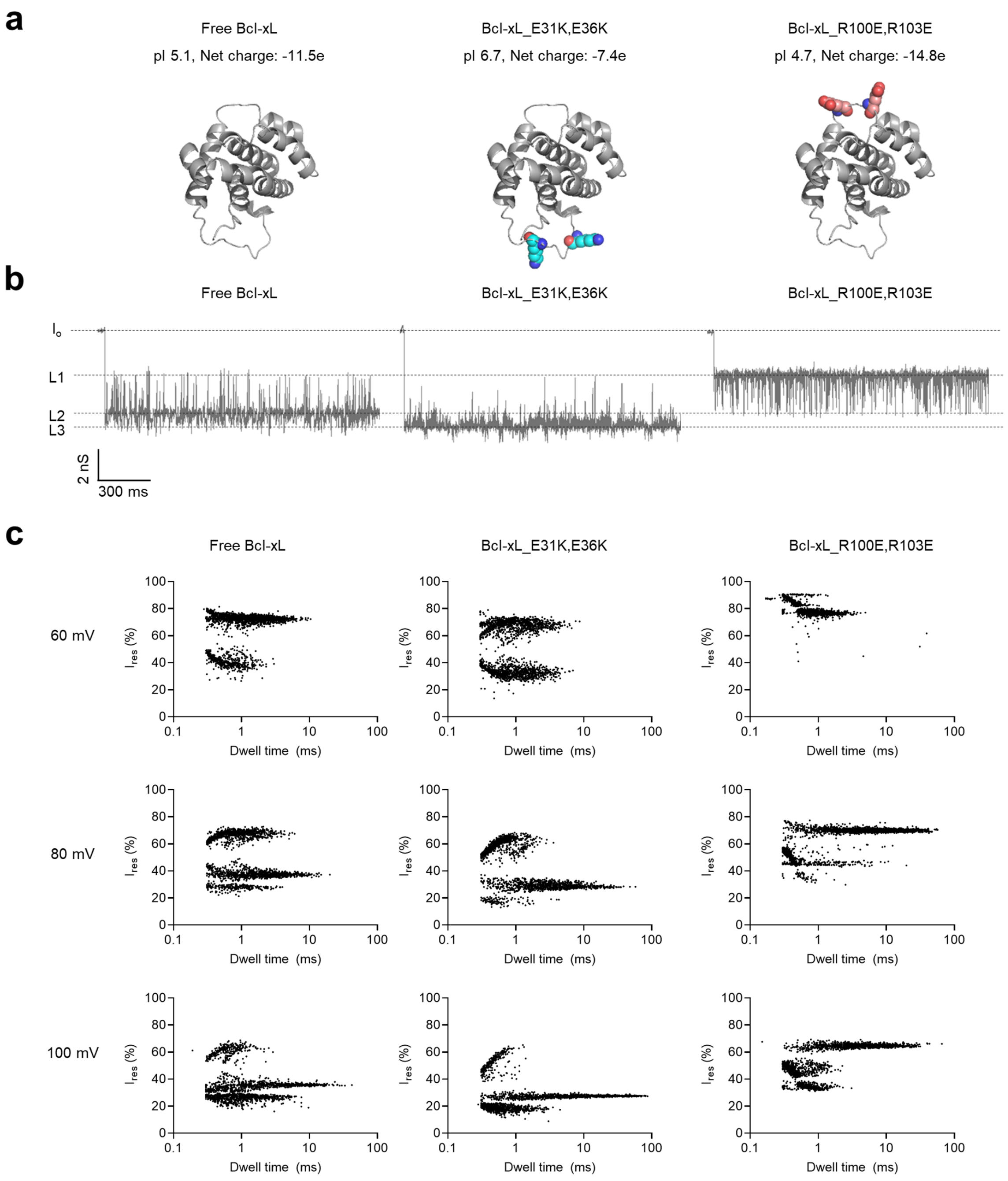

Supplementary Fig. 10: Distinctive current signal patterns of Bcl-xL and its mutants measured by YaxAB nanopores at different voltages. a. Bcl-xL and its variants (Bcl-xL_E31K,E36K as a positively charged mutant and Bcl-xL_R100E,R103E as a negatively charged mutant). The mutation sites are indicated as spheres (red and blue) in Bcl-xL structure (gray). **b.** Representative multi-level current blockades for each analyte measured at 100 mV. **c.** The scatter plot (I_{res} versus. dwell time) for wild-type Bcl-xL and its variants at various voltages (60, 80, and 100 mV). All electrical current was filtered with a low-pass Bessel filter at 1 kHz with a sampling rate of 100 kHz. All of statistical analysis was performed over 2,000 events.

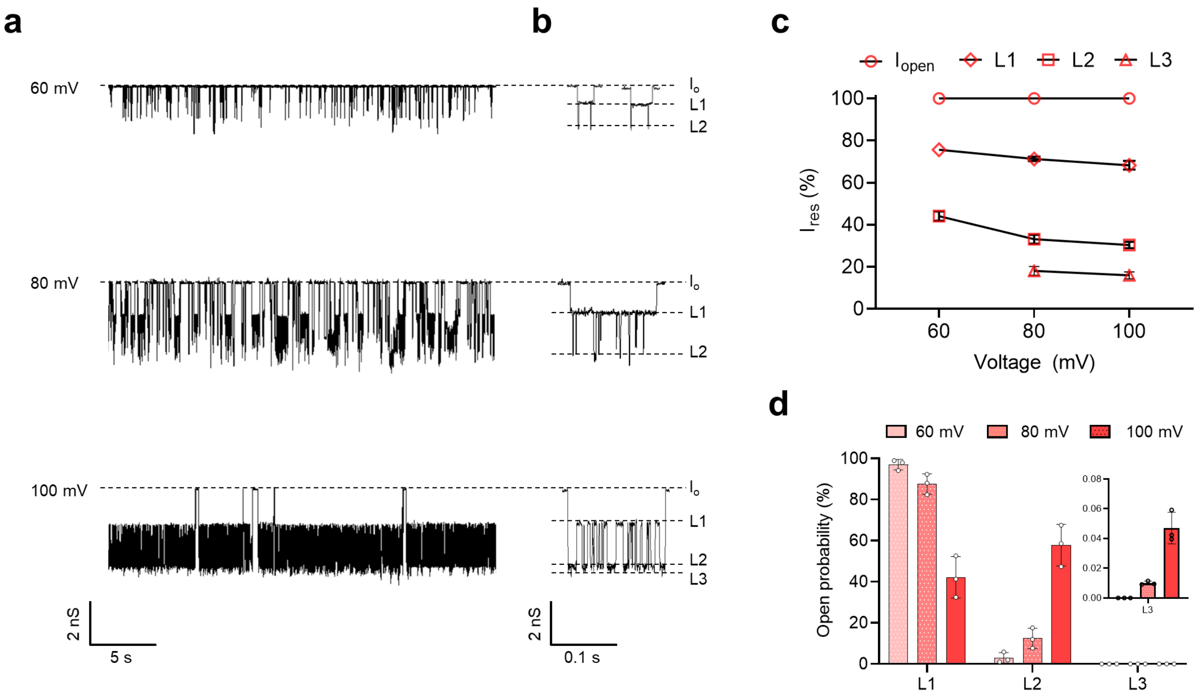

Supplementary Fig. 11: Current blockades analysis of Bcl-xL/Bak-BH3 complex using YaxAB nanopores at different voltages. a. Representative current traces of the Bcl-xL/Bak-BH3 complex trapped by a YaxAB nanopore with engaged voltages (60, 80, and 100 mV) and a 1 kHz filter. I_o indicates open current of the nanopore, and each ionic current levels are L1, L2, and L3, respectively. Residual current (I_{res}) gradually decreased with voltage increase until 100 mV. **b.** Representative nanopore events exhibiting vibration signals. **c.** Residual current of Bcl-xL/Bak-BH3 trapped by the YaxAB nanopore decreased with voltage increment (60 to 100 mV). Data are presented as mean ± SD, n = 3 independent replicates at each engaged voltage. The ratio of residual current of L1 to L3 levels at different voltage biases; L1 (75.67 ± 0.17%) and L2 (44.04 ± 2.21%) at 60 mV; L1 (71.28 ± 1.02%), L2 (33.10 ± 2.06%), and L3 (18.06 ± 2.11%) at 80 mV; L1 (68.24 ± 2.06%), L2 (30.32 ± 1.50%), and L3 (15.91 ± 1.63%) at 100 mV. Source data are provided as a Source Data file. **d.** Open probability of each peak level in Bcl-xL/Bak-BH3 as a function of engaged voltages. Data are presented as mean ± SD, n = 3 independent replicates at each engaged voltage. The open probability of L1 to L3 current levels at different voltage biases: L1 (96.99 ± 2.54%) and L2 (3.01 ± 2.54%) at 60 mV; L1 (87.56 ± 4.97%), L2 (12.43 ± 4.97%), and L3 (0.01 ± 0.001%) at 80 mV; L1 (42.36 ± 10.09%), L2 (57.59 ± 10.09%), and L3 (0.05 ± 0.01%) at 100 mV. Magnified open probability of L3 at three different voltages (inset). Source data are provided as a Source Data file.

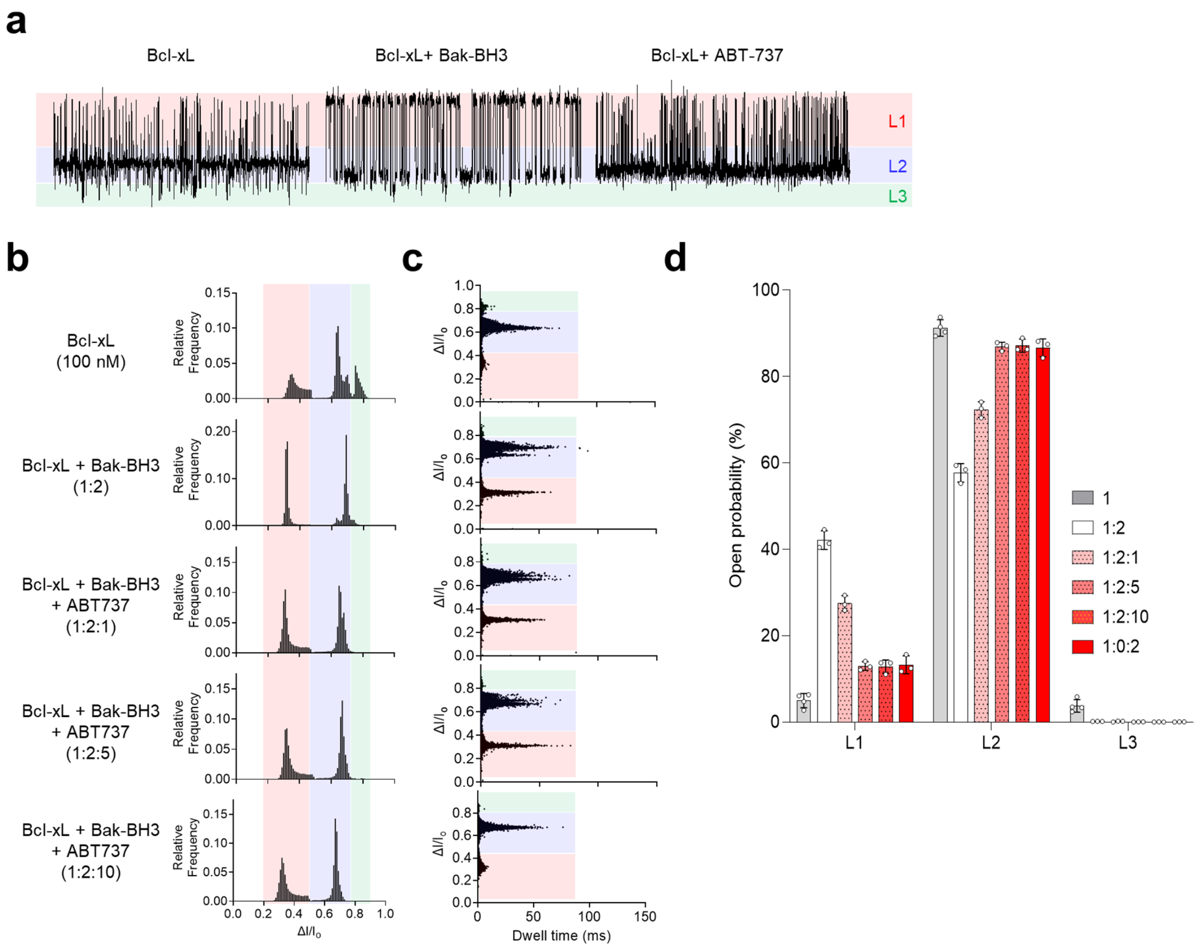

Supplementary Fig. 12: Current blockade-based analysis of Bcl-xL/Bak-BH3 interaction inhibition by ABT-737 using YaxAB nanopores. a. Representative current traces from Bcl-xL, Bcl-xL/Bak-BH3 complex (at the molar ratio of 1:2), and Bcl-xL/Bak-BH3 complex in the presence of ABT-737 (at the molar ratio of 1:2:10 Bcl-xL:Bak-BH3:ABT-737). All current blockades were categorized by three current levels (L1, L2, and L3). **b**. Statistical histograms of open probability of L1 to L3 current levels. The Bcl-xL/Bak-BH3 complex was titrated with ABT-737 at the molar ratios of 1:2:1, 1:2:5, and 1:2:10 to monitor the protein-protein interaction (PPI) inhibition by ABT-737. **c**. Scatter plots ($\Delta I/I_o$ vs. dwell time) of multi-level current blockades. All current traces were filtered using a Bessel (8-pole) 1 kHz filter. **d**. The Bcl-xL/Bak-BH3 (1:2) complex was titrated with ABT-737 at the molar ratios of 1:2:1, 1:2:5, and 1:2:10. Open probability of the Bcl-xL/ABT-737 (1:0:2) complex without Bak-BH3 was included for comparison. Data are presented as mean ± SD, n = 4 independent replicates for Bcl-xL; n = 3 independent replicates for the Bcl-xL/Bak-BH3 complex; n = 3 for Bcl-xL/Bak-BH3 complex in the presence of ABT-737. Source data are provided as a Source Data file. All electrical recordings were conducted in 1 M KCl at pH 7.5, with a 100 kHz sampling rate and a 10 kHz Bessel filter.

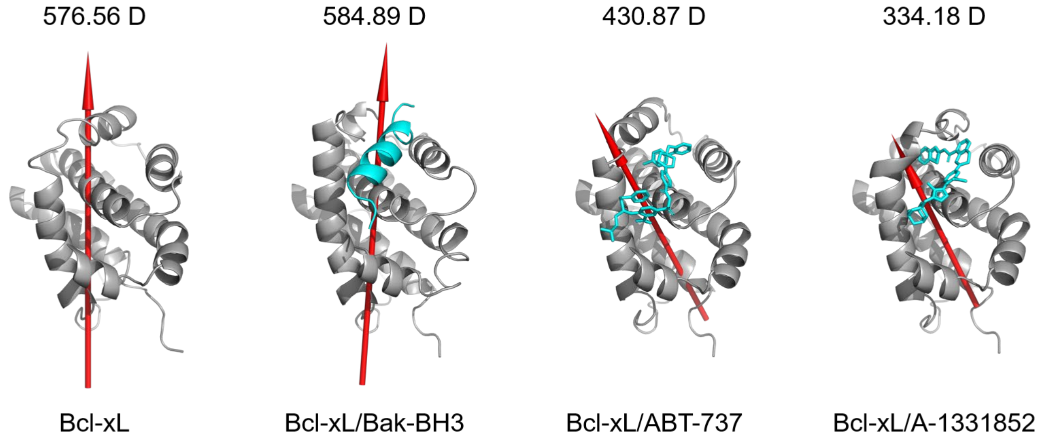

Supplementary Fig. 13: Orientation and strength of dipole moment for free Bcl-xL and three Bcl-xL complexes (Bcl-xL/Bak-BH3, Bcl-xL/ABT-737, and Bcl-xL/A-1331852). The dipole moments (Debye, D) were calculated by using a Discovery Studio software. The red arrows indicate the direction of the protein dipole. Bcl-xL and binders are shown in gray and cyan, respectively.

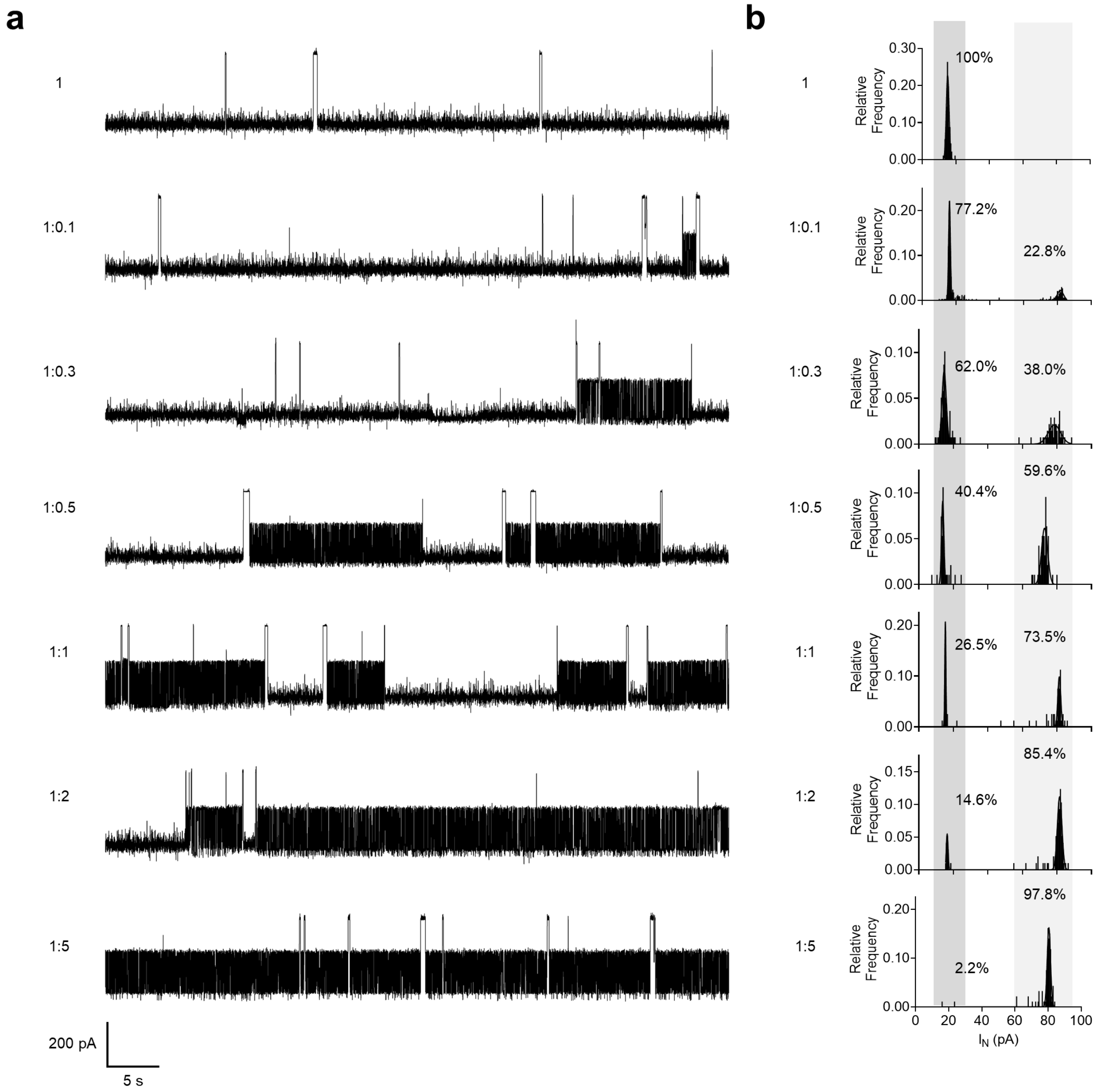

Supplementary Fig. 14: Current noise (I_N)-based analysis of nanopore events upon titration of Bcl-xL with Bak-BH3. a. Representative current traces obtained from titration of Bak-BH3 into Bcl-xL at the molar ratios of 1:0.1, 1:0.3, 1:0.5, 1:1, 1:2, and 1:5. **b**. Statistical histograms of I_N values for each titration point.

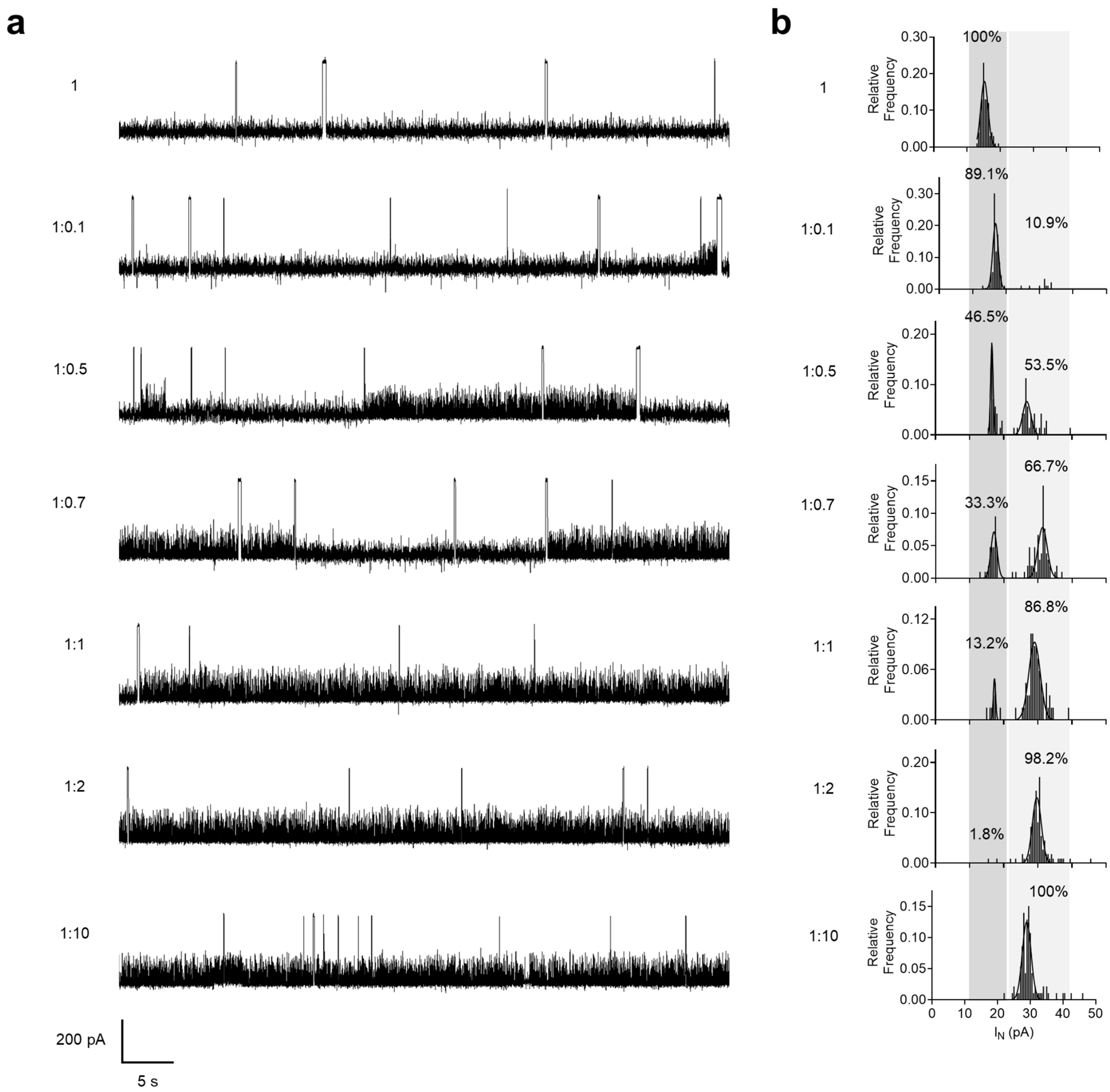

Supplementary Fig. 15: Current noise (I_N)-based analysis of nanopore events upon titration of Bcl-xL with ABT-737. a. Representative current traces obtained from titration of ABT-737 into Bcl-xL at the molar ratios of 1:0.1, 1:0.5, 1:0.7, 1:1, 1:2, and 1:10. **b**. Statistical histograms of I_N values for each titration point**.**

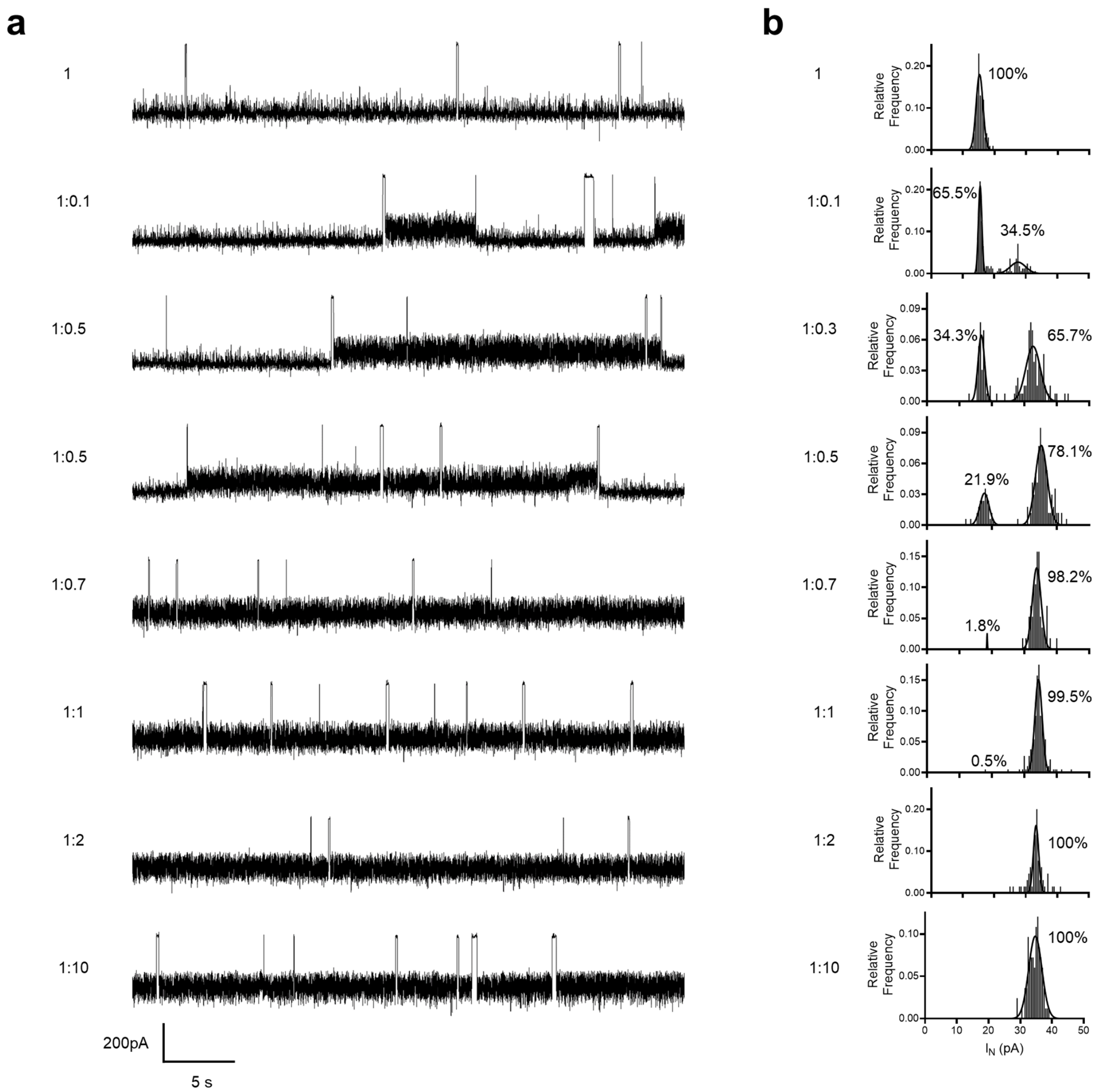

Supplementary Fig. 16: Current noise (I_N)-based analysis of nanopore events upon titration of Bcl-xL with A-1331852. a. Representative current traces obtained from titration of A-1331852 into Bcl-xL at the molar ratios of 1:0.1, 1:0.3, 1:0.5, 1:0.7, 1:1, 1:2, and 1:10. **b**. Statistical histograms of I_N values for each titration point**.**

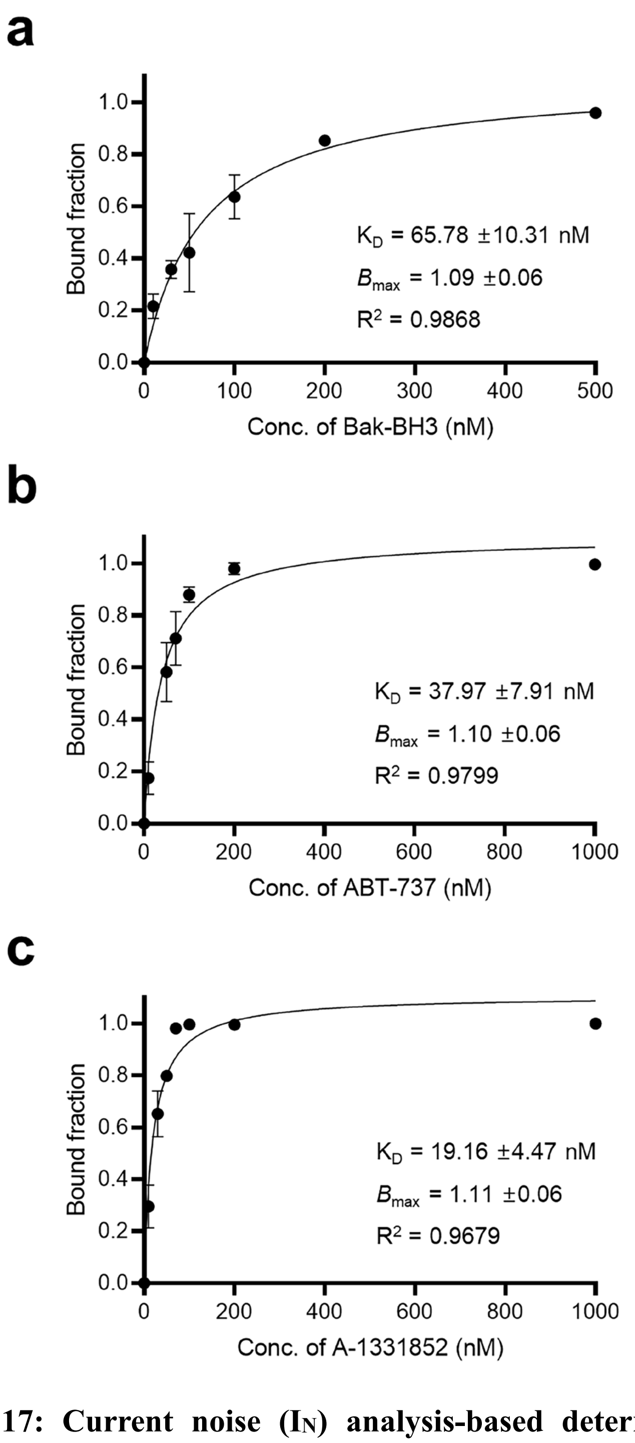

Supplementary Fig. 17: Current noise (I_N) analysis-based determination of Bcl-xL binding affinity for Bak-BH3 peptide, ABT-737, and A-1331852 using YaxAB nanopores. a-c. Hill diagrams (bound fraction vs. the concentration of ligand) of the Bcl-xL/Bak-BH3 complex (**a**), the Bcl-xL/ABT-737 complex (**b**), and the Bcl-xL/A-1331852 complex (**c**). Data are presented as mean ± SEM, n = 3 independent replicates. Source data are provided as a Source Data file. All measurements were performed by applying 100 mV to the *cis* chamber in 1 M KCl, 10 mM Tris-HCl (pH 7.5), and 1 mM EDTA, with sampling rate of 100 kHz using a 100 Hz Bessel filter. All analytes including Bcl-xL were added to the *cis* chamber.

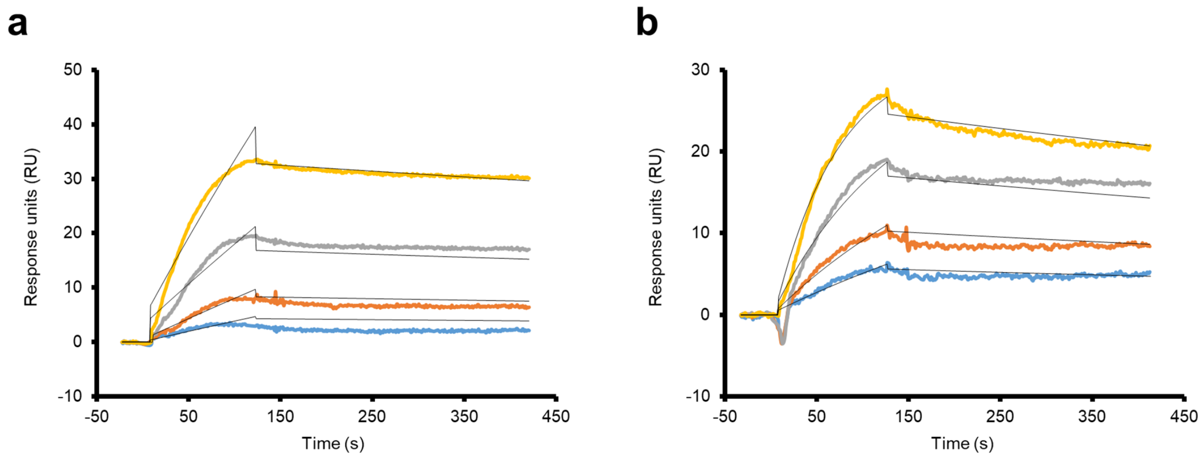

Supplementary Fig. 18. SPR analysis for interaction between Bcl-xL and small-molecule drugs. SPR sensorgrams are shown for binding of Bcl-xL with ABT-737 (**a**) and A-1331852 (**b**). The concentrations of small-molecule drugs used are 300 (yellow), 150 (gray), 75 (orange), and 37.5 nM (blue).

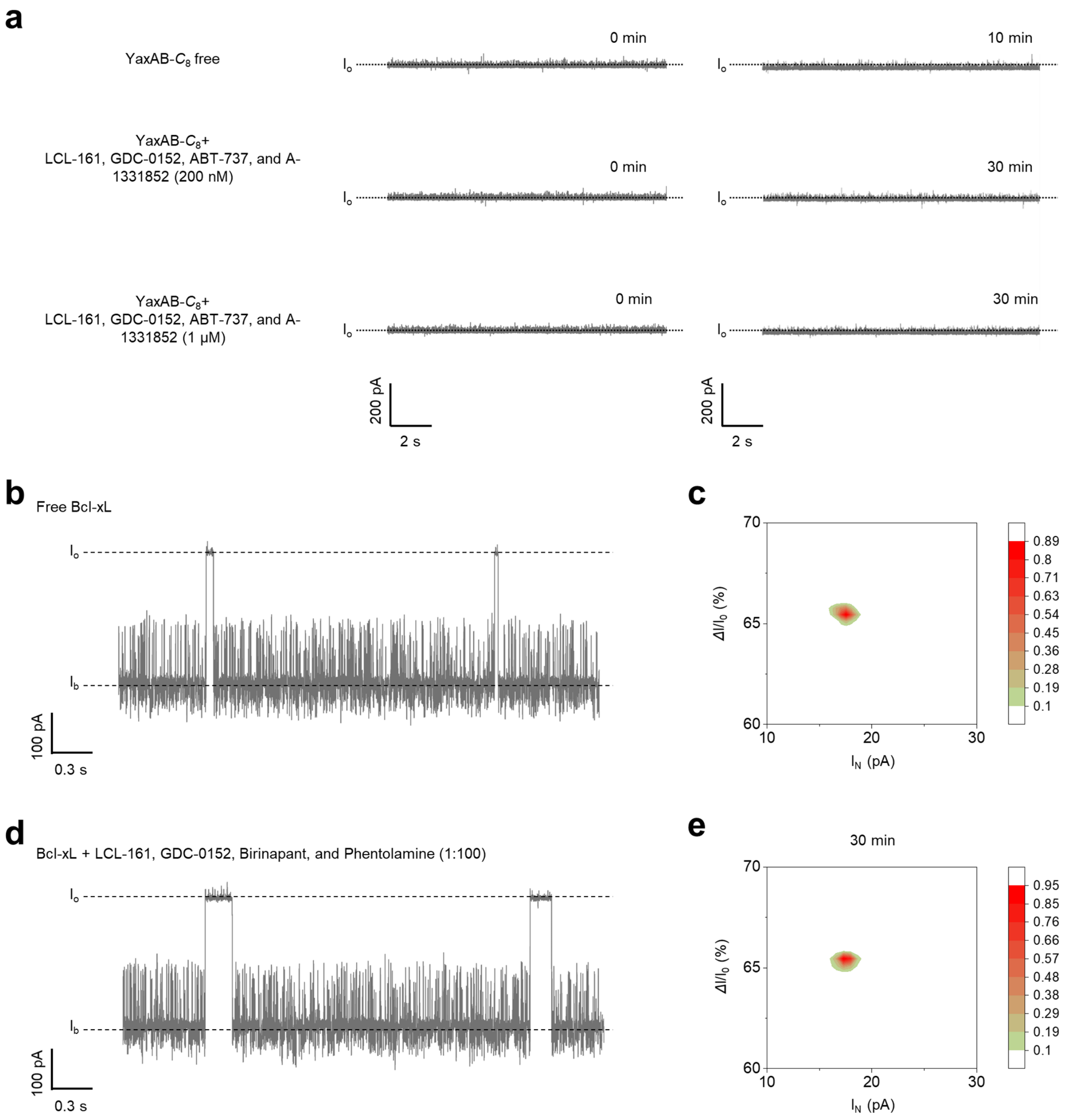

Supplementary Fig. 19. YaxAB nanopore measurements with non-specific small-molecule ligands. a. Representative current traces of free YaxAB-C_8 and YaxAB-C_8 with small-molecule non-binders (LCL-161, GDC-0152, ABT-737, and A-1331852) at 200 nM and 1 μM. **b-e.** Representative multi-level current blockades (**b, d**) and 2D $\Delta I/I_o$-versus-I_N density contour plot of free Bcl-xL and Bcl-xL with small-molecule non-binders (LCL-161, GDC-0152, Birinapant, and Phentolamine) at a molar ratio of 1:100 (**c, e**).

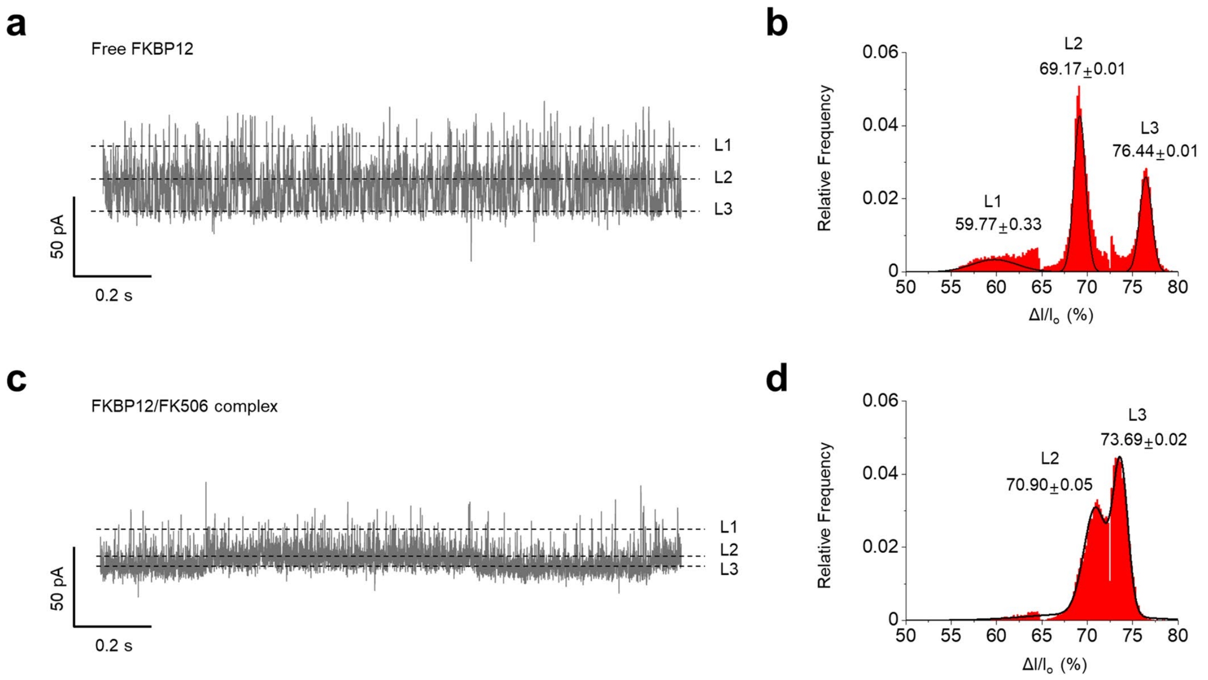

Supplementary Fig. 20: Detection of FKBP12/FK506 interaction using YaxAB nanopores. **a-b.** Multi-level current blockades (**a**), and histogram of $\Delta I/I_o$ of free FKBP12 (**b**). **c-d.** Multi-level current blockades (**c**), and histogram of $\Delta I/I_o$ of FKBP12/FK506 complex (**d**).

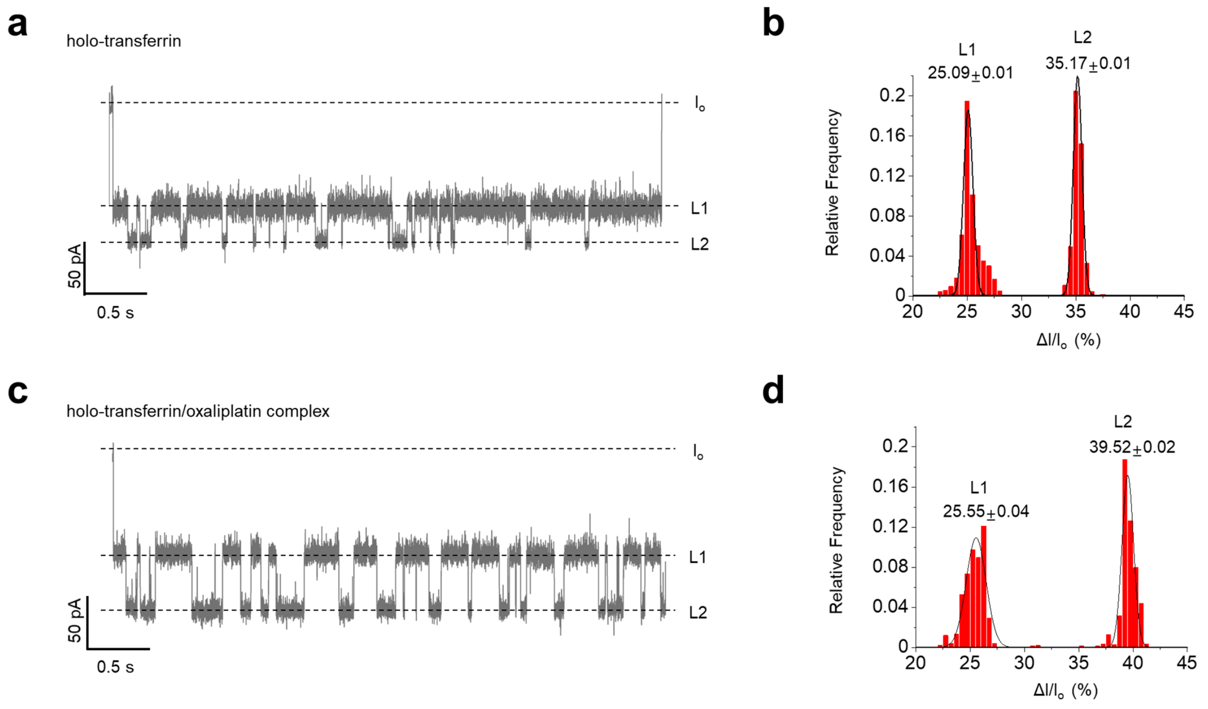

Supplementary Fig. 21: Detection of holo-transferrin/oxaliplatin interaction using YaxAB nanopores. a-b. Representative current traces showing multi-level current blockades (L1 and L2) (**a**), and histogram of $\Delta I/I_o$ of free holo-transferrin (**b**). **c-d.** Representative current traces showing multi-level current blockades (L1–L2) (**c**), and histogram of $\Delta I/I_o$ of the holo-transferrin/oxaliplatin complex (**d**).

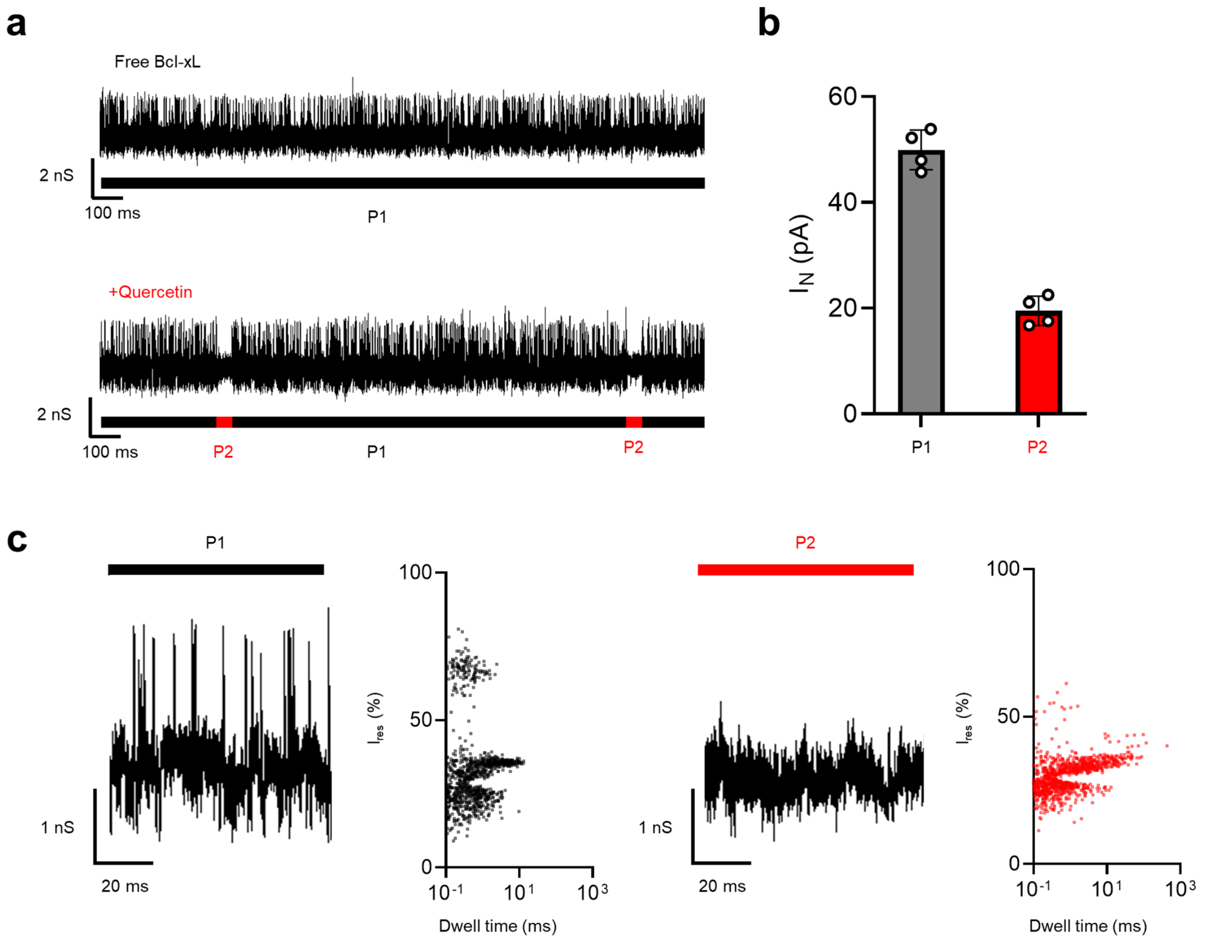

Supplementary Fig. 22: Current blockade and noise analyses of Bcl-xL/Quercetin interactions using YaxAB nanopores. a. Representative current traces for free Bcl-xL and the addition of Quercetin. Current signal pattern P1 (black) is temporally transformed to P2 in the presence of Quercetin (red). **b.** Current noise (I_N) values of P1 and P2. Data are presented as mean ± SD, n=4 independent replicates for Bcl-xL in the presence of Quercetin. The mean value of I_N: P1, 49.17 ± 4.21 pA; P2, 18.95 ± 3.12 pA. Source data are provided as a Source Data file. **c.** Representative zoom-in current traces and corresponding scatter plot (I_{res} vs. dwell time) of Bcl-xL (P1) and the addition of Quercetin (P2). All current traces were recorded at +100 mV in 1 M KCl, 10 mM Tris-HCl, 1 mM EDTA, pH 7.5, with 1 μM Bcl-xL in the presence of Quercetin (at 1:20 molar ratio).

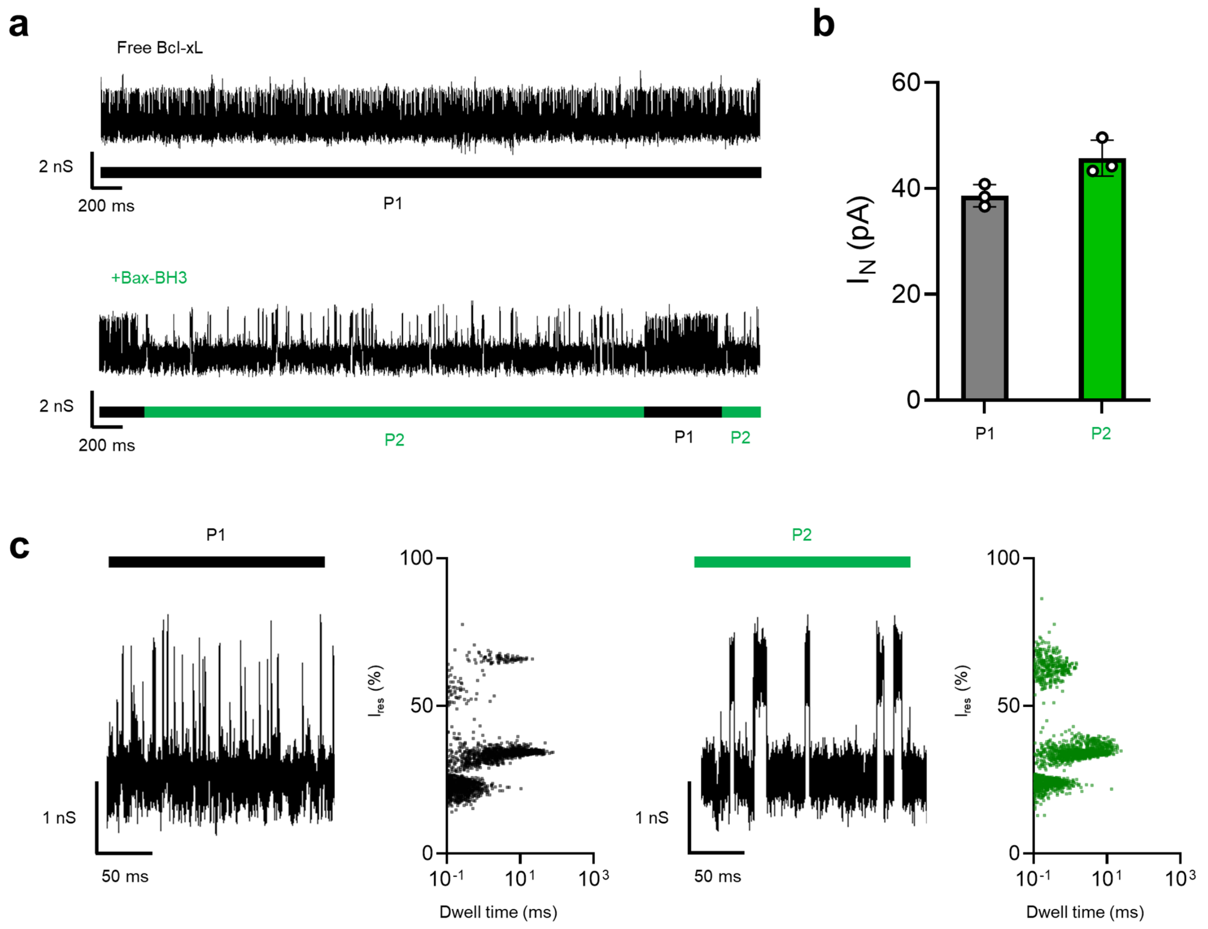

Supplementary Fig. 23: Current blockade and noise analyses of Bcl-xL/Bax-BH3 interactions using YaxAB nanopores. a. Representative current traces for free Bcl-xL and the addition of Bax-BH3 peptide. Current signal pattern P1 (black) is temporally transformed to P2 in the presence of Bax-BH3 peptide (green). **b.** Current noise (I_N) values of P1 and P2. Data are presented as mean ± SD, n = 3 independent replicates for Bcl-xL in the presence of Bax-BH3 peptide. The mean value of I_N: P1, 38.62 ± 2.09 pA; P2, 45.72 ± 3.39 pA. Source data are provided as a Source Data file. **c.** Representative current traces and corresponding scatter plot (I_{res} vs. dwell time) of Bcl-xL (P1) and the addition of Bax-BH3 peptide (P2). All current traces were recorded at +100 mV in 1 M KCl, 10 mM Tris-HCl, 1 mM EDTA, pH 7.5, with 1 μM Bcl-xL in the presence of Bax-BH3 peptide (at 1:100 molar ratio).

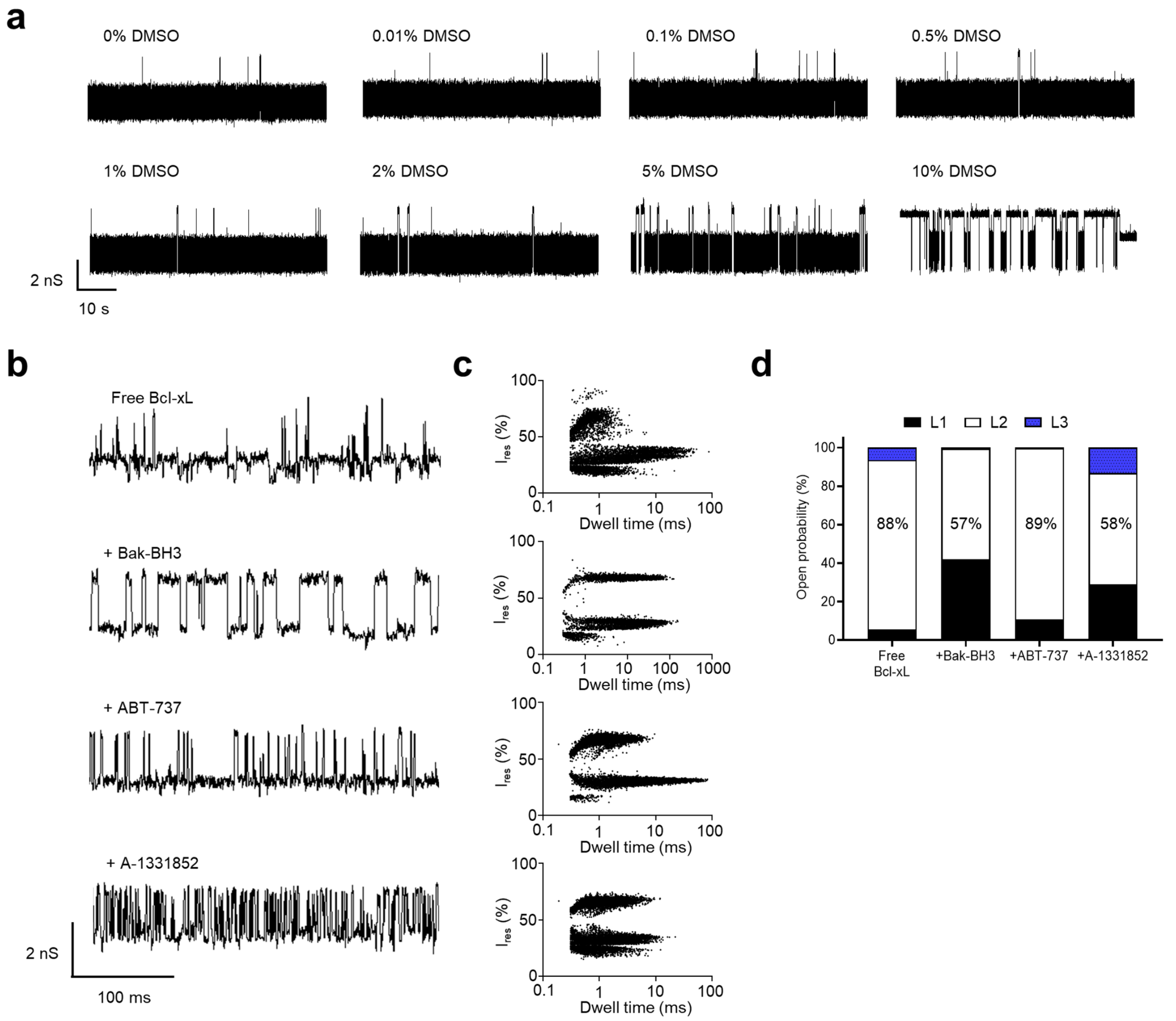

Supplementary Fig. 24: YaxAB nanopore measurements in the presence of DMSO. a. Representative current traces of Bcl-xL in the presence of DMSO at various concentrations (0 to 10 %). **b-c.** Representative multi-level current blockades (**b**) and scatter plots (I_{res} vs. dwell time) of Bcl-xL and three complexes, Bcl-xL/Bak-BH3, Bcl-xL/ABT-737, and Bcl-xL/A-1331852 complexes (**c**). **d.** Stacked columns for open probability of L1-L3 levels measured from current blockades of free Bcl-xL and its complexes.

Supplementary Table 1. Current-voltage (I-V) curves for YaxAB nanopores in symmetric salt conditions at pH 7.5*

Voltage	YaxAB-C_{10}, 1 M KCl		YaxAB-C_9, 1 M KCl		YaxAB-C_8, 1 M KCl	
(mV)	Current (pA)	S.D.	Current (pA)	S.D.	Current (pA)	S.D.
-100	-1165.8	23.8	-898.7	21.7	-639.1	9.0
-80	-928.0	28.3	-707.6	11.8	-501.8	5.3
-60	-687.7	18.1	-526.0	8.6	-369.3	3.8
-40	-453.6	16.3	-347.2	5.5	-241.4	1.2
-20	-223.4	11.2	-172.1	2.9	-117.9	0.7
0	0.0	0.0	0.0	0.0	0.0	0.0
20	227.2	4.0	167.2	3.2	113.2	1.0
40	450.6	7.4	328.5	5.7	220.9	2.9
60	668.9	10.7	484.9	9.4	321.4	3.5
80	881.0	14.2	633.5	11.9	416.6	5.5
100	1086.5	16.9	776.0	17.2	506.1	6.4

* The buffer contained 1 M KCl, 10 mM Tris-HCl (pH 7.5), and 1 mM EDTA. Each current data represents the average value. Standard deviations are based on data in triplicate. Source data are provided as a Source Data file.

Supplementary Table 2. Current-voltage (I-V) curves for YaxAB nanopores in asymmetric salt conditions at pH 7.5*

Voltage	YaxAB-C_{10}, 2 M KCl in *trans*		YaxAB-C_9, 2 M KCl in *trans*		YaxAB-C_8, 2 M KCl in *trans*	
(mV)	Current (pA)	S.D.	Current (pA)	S.D.	Current (pA)	S.D.
-50	-646.9	44.9	-487.3	13.4	-349.3	34.8
-40	-522.8	34.7	-395.6	7.7	-282.5	28.4
-30	-402.3	22.8	-306.8	3.7	-221.8	21.1
-20	-282.9	14.0	-218.9	2.8	-161.2	15.7
-10	-170.5	11.5	-133.2	2.9	-105.4	9.0
0	-53.2	9.4	-49.7	3.8	-51.0	4.0
10	61.4	9.2	31.0	4.9	1.7	2.3
20	172.9	11.0	108.8	6.5	51.2	6.6
30	280.5	13.6	184.2	8.2	99.0	10.2
40	388.9	11.8	255.8	9.8	144.4	15.1
50	488.3	18.1	326.1	11.0	187.4	18.8

* The buffer contained either 2 M or 0.5 M KCl in 10 mM Tris-HCl (pH 7.5) and 1 mM EDTA. Each current data represents the average value. Standard deviations are based on data in triplicate. Source data are provided as a Source Data file.

Supplementary Table 3. Ion selectivity of YaxAB nanopores

Pore type	YaxAB-C_{10}	YaxAB-C_9	YaxAB-C_8
Reversal potential (mV)	5.48 ± 0.14	7.65 ± 0.70	11.69 ± 0.14
P_{K^+}/P_{Cl^-}	1.47 ± 0.01	1.72 ± 0.09	2.34 ± 0.03

Data are presented as mean ± SD, n = 3 independent replicates. Source data are provided as a Source Data file.

Supplementary Table 4. Molecular properties of Bcl-xL and its complexes

Molecule	M.W. (Da)	Excluded volume (Å^3)	Dipole moment (D)
Bcl-xL	20,781.0	24,301	576.56
Bcl-xL/Bak-BH3*	22,505.9	26,306	584.89
Bcl-xL/ABT-737	21,594.4	24,021	430.87
Bcl-xL/A-1331852	21,439.8	23,756	334.18

* PDB code: 1BXL
